# Identification of Epigenetic Regulatory Networks of Gene Methylation–miRNA–Transcription Factor Feed-Forward Loops in Basal-like Breast Cancer

**DOI:** 10.3390/cells14161235

**Published:** 2025-08-10

**Authors:** Larissa M. Okano, Alexandre L. K. de Azevedo, Tamyres M. Carvalho, Jean Resende, Jessica M. Magno, Bonald C. Figueiredo, Tathiane M. Malta, Mauro A. A. Castro, Luciane R. Cavalli

**Affiliations:** 1 Research Institute Pelé Pequeno Príncipe, Faculdades Pequeno Príncipe, Curitiba 80250-060, PR, Brazil; 2 Genetics Department, Federal University of Paraná, Curitiba 80060-000, PR, Brazil; 3 School of Pharmaceutical Sciences, University of São Paulo, Ribeirão Preto 14040-903, SP, Brazil; 4 Bioinformatics and Systems Biology Laboratory, Federal University of Paraná, Curitiba 81520-260, PR, Brazil; 5 Oncology Department, Lombardi Comprehensive Cancer Center, Georgetown University, Washington, DC 20057, USA

**Keywords:** DNA methylation, genes, distal probes, basal-like breast cancer, transcription factors, miRNA, feed-forward loops, regulatory epigenetic networks

## Abstract

Basal-like breast cancer (BLBC) is associated with poor prognosis, high recurrence rates, and limited therapeutic options, largely due to its molecular heterogeneity and complexity, which include epigenetic alterations. This study investigated epigenetic regulatory networks in BLBC by analyzing DNA methylation in distal cis-regulatory regions and its impact on genes, transcription factors (TFs), and microRNAs (miRNAs) expression. Data from TCGA were processed using the ELMER and DESeq2 tools to identify differentially methylated regions and differentially expressed genes, TFs, and miRNAs. The FANMOD algorithm was used to identify the regulatory interactions uncovering the feed-forward loops (FFLs). The analysis identified 110 TF-mediated FFLs, 43 miRNA-mediated FFLs, and five composite FFLs, involving 18 hypermethylated and 32 hypomethylated genes, eight upregulated and nine downregulated TFs, and 21 upregulated and seven downregulated miRNAs. The TF-mediated FFLs major regulators involved the *AR*, *EBF1*, *FOS*, *FOXM1*, and *TEAD4* TFs, while key miRNAs were miR-3662, miR-429, and miR-4434. Enriched pathways involved cAMP, ErbB, FoxO, p53, TGF-beta, Rap1, and Ras signaling. Differences in hallmark gene set categories reflected distinct methylation and miRNA expression profiles. Overall, this integrative analysis mapped the intricate epigenetic landscape of BLBC, emphasizing the role of FFLs as regulatory motifs that integrate DNA methylation, TFs, and miRNAs in orchestrating disease’s development and progression and offering potential targets for future diagnostic and therapeutic strategies.

## 1. Introduction

The basal-like breast cancer (BLBC) subtype represents 10% of breast cancer cases overall [1]. Its incidence is notably elevated among young women, those with a *BRCA1* gene variant mutation, and those of African descent [2,3]. BLBC presents clinically aggressive phenotypes, which include the development of distant metastasis shortly after diagnosis. These tumors also exhibit high inter- and intratumoral molecular heterogeneity, challenging the selection of effective treatment strategies. This inherent aggressiveness and heterogeneous nature of BLBC collectively confer to the patient’s poor prognosis and a diminished response to conventional therapeutic interventions [4,5].

Epigenetic alterations, including histone modifications, changes in DNA methylation patterns, and microRNAs (miRNAs) expression, are associated with the development and progression of breast cancer, including the BLBC [4,5,6]. DNA methylation exerts a direct impact on gene transcription, which can be mediated by the regulation of gene enhancers and silencers located in distal upstream and downstream regulatory regions, as well as, within introns [7,8,9]. Studies have demonstrated that these distal regulatory regions are associated with gene expression profiles and often exhibit differential DNA methylation patterns that are not observed in promoter regions [8,9]. These regions contribute to gene regulation through long-range chromatin interactions and can act in a cell type or context-specific manner, offering key insights into tumor-specific transcriptional regulation. In breast cancer, including the BLBC, aberrant methylation in distal elements has been linked to the activation or repression of critical oncogenes and tumor suppressors [8,9]. Thus, investigating methylation changes in these regions may reveal regulatory mechanisms and epigenetic drivers that remain undetected when focusing solely on promoter methylation.

In addition, these alterations can also impact the binding of transcription factors (TFs) to their target sites, particularly in methylated regions [10]. The methylated cytosine–guanine dinucleotides (CpGs) can repress the TF binding within their motifs and impact their regulatory action [11,12,13]. Studies have also shown that TF binding to methylated regions can also induce demethylation [14,15,16] and that the TF sensitivity to DNA methylation depends on the position of methylated CpGs within their binding motifs [17,18,19].

DNA methylation can also regulate miRNA biogenesis [20]. When the flanking regions of miRNA coding sequences become significantly methylated, an increase in miRNA expression is often observed. These methylated miRNAs are also more likely to be involved in cancer-related phenotypes compared to those originating from unmethylated coding regions [20]. In addition, extensive research on cancer, including BLBC, has described the intricate crosstalk between DNA methylation and miRNA expression [21,22,23,24]. Specifically, methylation in miRNA promoter regions inhibits its transcription, while miRNAs can target transcripts to regulate proteins involved in DNA methylation [25,26].

Furthermore, substantial research has provided valuable information on the crosstalk of miRNAs, and other non-coding RNAs and TFs in the context of cancer, notably encompassing regulatory mechanisms, such as feed-forward loops (FFLs) [27,28,29,30,31,32,33]. The FFLs may be composed of two regulatory factors, such as miRNA and TF [34,35]. The FFLs can be classified into three types based on the regulatory interactions between the miRNA and TF: the FFL mediated by TF is defined when the TF regulates the gene and miRNA at the transcriptional level, with the miRNA subsequently repressing the gene at the post-transcriptional level; second, the FFL mediated by miRNA, characterized by the miRNA repressing the gene and TF, while TF regulates the gene; and finally, the composite FFL, where the miRNA and TF mutually regulate each other, forming a feedback loop, and both regulating a common target gene.

In this study, the primary aim was to identify DNA methylation distal probes in BLBC and determine their impact on the transcriptional expression of genes, TFs, and miRNAs. The regulatory networks, through these interactions, along with the three-node FFLs characterization, were systematically evaluated for their impact on biological signaling pathways and clinical significance in BLBC.

## 2. Materials and Methods

### 2.1. General Study Design

**Figure 1:**
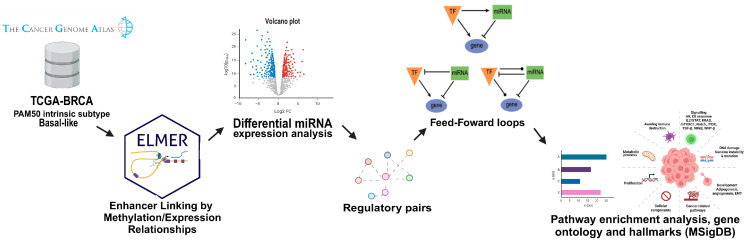
Workflow of the computational analysis of the study.

To achieve the main aim of the study, a comprehensive computational pipeline applying R language was used following the workflow presented in Figure 1. Briefly, the transcriptional expression profiles (RNA-seq and microRNA-seq) and DNA methylation of basal-like breast cancer (BLBC) and non-tumor breast samples were retrieved from The Cancer Genome Atlas (TCGA)-Breast Cancer (BRCA) database [36] using the TCGABiolinks/R (v.2.23.9) package(University of São Paulo, Ribeirão Preto, SP, Brazil; University of Miami Miller School of Medicine, Miami, FL, USA) [37,38]. Subsequently, the ELMER (v.2.21.0) package (Cedars-Sinai Medical Center, Los Angeles, CA, USA; University of São Paulo, Ribeirão Preto, SP, Brazil) [39], designed to use DNA methylation and gene expression information to infer regulatory elements and TFs network, was used to identify the differentially methylated distal probes between the two breast sample groups. Differentially expressed (DE) miRNAs and TFs between these groups were identified using the DESeq2 (v.1.34.0) package (Dana-Farber Cancer Institute, Boston, MA, USA; Harvard School of Public Health, Boston, MA, USA; Max Planck Institute for Molecular Genetics, Berlin, Germany) [40].

The regulatory pairs encompassing gene–gene, miRNA–gene, miRNA-TF, TF-TF, TF–gene, and TF-miRNA) were determined by the multiMiR [41], ENCODE, and CHIP-X (CHEA) [42,43,44] tools, with the corresponding feed-forward loops (FFLs) by the FANMOD algorithm [45]. Further, pathway enrichment analysis (PEA) was conducted using the microT-CDS (v.5.0), Targetscan (v.7.0), and Tarbase (v.8.0) available at Diana Tools mirPath (v.4.0) [46] and REACTOME pathways [47,48]. Gene Ontology (GO) terms and Hallmark genes set collection were analyzed using the Molecular Signatures Database (MSigDB; v.2023.1) [49,50]. The intersection of the three databases was performed to identify the most common Kyoto Encyclopedia of Genes and Genomes (KEGG) pathways.

### 2.2. Data Collection

DNA methylation data derived from the Illumina Infinium Human Methylation 450 K microarray, transcriptional gene expression profiles (RNA-seq (level 3 data), and microRNA expression profiles (microRNA-seq) were retrieved from The Cancer Genome Atlas (TCGA)—Breast Cancer (BRCA) collection using the TCGABiolinks/R (v.2.23.9) package [37,38]. The data was obtained from breast cancer patients with the BLBC subtype (RNA-seq (n = 134), DNA methylation (n = 134) and miRNA-seq (n = 129)) defined by the PAM50 analysis [51,52], and from non-tumor breast samples (n = 84) for comparison analysis.

### 2.3. Epigenetic Profiles

The comparison between the selected BLBC samples and non-tumor breast samples was performed using the Enhancer Linking by Methylation/Expression Relationships (ELMER) package [39]. This package is specifically designed to use DNA methylation levels for the identification of enhancer elements in the genome associated with distal regions. It correlates these findings with gene expression to infer the presence of TF binding motifs and the expression of TF genes. Only samples that presented both gene expression and DNA methylation data were included in these analyses. The following parameters were used for the ELMER analysis: get.diff.meth (sig.diff) = 0.3, get.diff.meth (*p*_value) = 0.01, get.pair (raw.pvalue) = 0.05, get.enriched.motif (lower.OR) = 1.1, get.enriched.motif (min.incidence) = 10.

### 2.4. Differential Genes, TFs, and miRNAs Expression Analysis

The differentially expressed miRNAs and TFs were identified from the microRNA-seq data and RNA-seq data, respectively. The DESeq2 package [40], was used to compare the two groups of samples (BLBC vs. non-tumor breast tissue) according to the miRNAs and TFs expression. The Wald test was used with Log2FoldChange (LogFC) < −0.58 threshold adopted for downregulated expression and LogFC > 0.58 for upregulated expression, with a significant *p*-value < 0.05.

### 2.5. Identification of Gene–TF–miRNA Epigenetic Regulatory Networks and FFLs

The construction of regulatory pairs (TF–gene, miRNA–gene, TF-miRNA, and miRNA-TF), was performed by the multiMIR (v.1.16.) [41] database and the ENCODE and CHIP-X (CHEA) datasets available at the HARMONIZOME databank [42,43,44].

**Figure 2:**
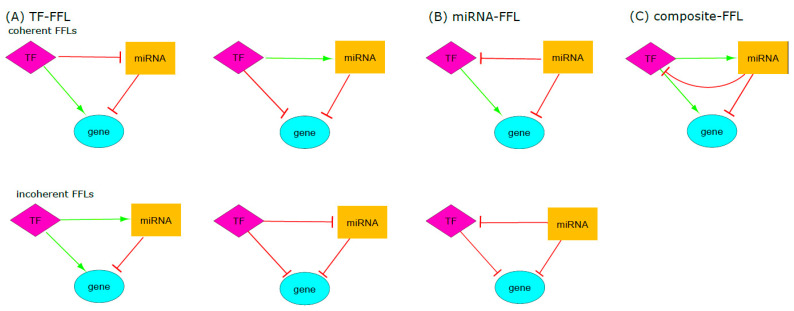
FFLs subtypes. (**A**) TF-FFLs: TF regulates the gene and miRNA at the transcriptional level, and miRNA represses the gene. (**B**) miRNA-FFLs: miRNA represses the gene and TF, while TF activates the gene. (**C**) composite FFL: miRNA and TF mutually regulate each other to form a feedback loop, and both regulate the common gene target. Coherent FFLs: two paths regulate the target gene with the same effects (either activation or repression). Incoherent FFLs: the target is regulated by two opposite paths. Nodes: diamonds = TFs; rectangles = miRNAs; ovals = genes; green arrows = activation; red T-shaped arrows = repression (Adapted: Zhang et al., 2013 [35]).

To determine the regulatory relationship (activation/repression), within each regulatory pair considering the differentially methylated/expressed genes, TFs, and miRNAs, Pearson correlation was used (https://hbiostat.org/R/Hmisc/ (accessed on 30 July 2023)). The feed-forward loops (FFLs) were identified by the FANMOD algorithm of random network generation [45]. Only FFLs composed of 3 nodes (gene, TF, and miRNA) were considered in the analysis. The FFLs can be of distinct subtypes: coherent, where two paths regulate the target gene with the same effects (activation or repression), and incoherent, in which the target gene is regulated by two opposite paths [35] (Figure 2).

### 2.6. Pathway Enrichment Analysis and Gene Ontology

The microT-CDS, TargetScan (v.8.0), and Tarbase (v.8.0) datasets of the DIANA-miRPath (v.4.0) database [46] were accessed to determine the targets of the miRNAs differentially expressed (DE) between the breast sample groups and the Kyoto Encyclopedia of Genes and Genomes (KEGG) to identify the corresponding gene pathways affected. The REACTOME pathways [48,49] and Gene Ontology (GO) terms and Molecular Signatures Database (MsigDB; v.2023.1) [50,51] were used to identify the pathways and processes related with DE genes, considering the GO categories Biological Process (BP), Cellular Component (CC), and Molecular Function (MF). A FDR-value < 0.05 was applied to identify significant associations.

### 2.7. Pathway Enrichment Analysis—Hallmark Gene Set Collection Analysis

The Molecular Signatures Database (MsigDB (v.2023.1)) was used to identify the specific biological pathways associated with the genes, TFs, and miRNAs identified composing the FFLs. This database uses a “hallmark gene set collection”, composed of 50 Hallmark names (e.g., signaling pathways, biological process, etc.), associated with large gene sets, which classifies the genes, TFs, and miRNAs into eight process categories: cellular component, development, DNA damage, immune, metabolic, pathway, proliferation, and signaling [50]. This classification provides a categorization of the biological entities involved in the FFLs, providing insights into their functional roles across diverse biological processes and signaling pathways. 

## 3. Results

### 3.1. Hypo-And Hyper-Methylated Probe–Gene Pairs and miRNAs and TFs Master Regulators (MRTFs)

The TCGA search for BLBC cases resulted in 134 cases with available DNA methylation and RNA-seq data and in 129 cases with microRNA-seq data. For the non-tumor breast cancer samples, 84 cases were obtained with available DNA methylation and RNA-seq data and 56 with microRNA-seq data.

**Figure 3:**
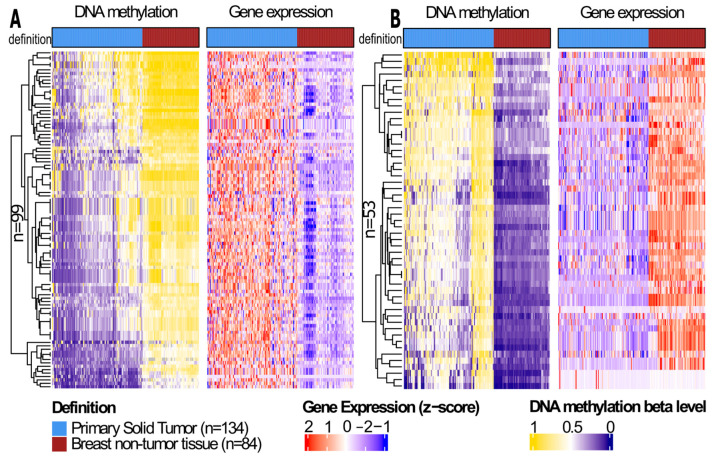
Heatmap of probe–gene pairs with a negative correlation between DNA methylation and gene expression between basal-like breast cancer (BLBC) primary solid tumor samples (n = 134) and breast non-tumor tissue samples (n = 84) using ELMER/R (v.2.21.0) package. (**A**) Heatmap of hypomethylation genes involving 99 probe–gene pairs and (**B**) of hypermethylation genes involving 53 probe–gene pairs.

The analysis of the epigenetically altered genes in BLBC tumors compared to the non-tumor breast cancer samples revealed 152 differentially methylated genes. Among these, 99 were hypomethylated (Figure 3A), and 53 were hypermethylated (Figure 3B), demonstrating a negative correlation between DNA methylation and gene expression. Detailed information on these genes, including corresponding *p* values and probe–gene pairs distance, are presented in Table S1A,B.

In the miRNA expression analysis, 500 miRNAs (317 upregulated and 183 upregulated) were observed differentially expressed in the comparison of the BLBC and non-tumor breast sample groups (Table S2). In the subsequent analysis, the enriched motifs belonging to probe–gene pairs were identified (Table S3A,B). The top 10 motifs with the highest number of probe pairs were: *AP2D*, *KLF3*, *MLX*, *PATZ1*, *SP1*, *SP2*, *SP3*, *ZF64A*, *ZFX*, and *ZN263*. The most common TF’s families were factors with multiple dispersed zinc fingers, three-zinc finger Kruppel, thyroid hormone receptor, *ETS*, *FOX*, *NK*, *SOX*, *TAL*, and *HOX*-related factors.

For each motif, the pipeline systematically computed the expression of each gene with average DNA methylation across all probes using probe–gene pairs. This allows the identification of possible TFs associated with the identified motifs. Finally, the MRTFs around CpG loci were identified. These binding sites were specifically linked to the expression levels correlated with the DNA methylation known to bind these enriched motifs. Through this comprehensive analysis, a total of 11 down- (*AR*, *DACH1*, *EBF1*, *EGR1*, *ERG*, *ESR1*, *FOS*, *KLF2*, *KLF4*, *PPARG*, *SOX17*, and *TAL1*) and 14 up-regulated (*CBX2*, *DMRT1*, *E2F1*, *FOXM1*, *GBX2*, *KLF1*, *MYBL1*, *MYBL2*, *PAX6*, *SALL4*, *SOX11*, *TCF21*, *TEAD4*, and *WT1*) MRTFs were identified.

### 3.2. Epigenetic Gene–TF–miRNA Networks and Formed FFLs

**Figure 4:**
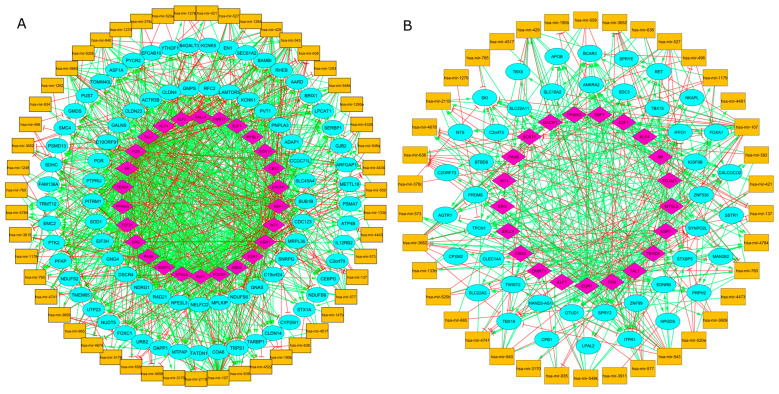
Epigenetics networks of genes, TFs, and miRNAs, of the BLBC subtype based on gene (**A**) hypomethylation and (**B**) hypermethylation. Blue circles = genes; orange boxes = miRNAs; pink diamonds = TFs, green arrows = gene expression activation; red T-shaped arrows = gene expression inhibition.

The regulatory pairs between genes, TF, and miRNA were identified according to the gene methylation profile (hypomethylated and hypermethylated) and used to construct the regulatory networks. A potential regulatory relationship (activation or repression) was observed between 637 TF–GENE, 127 TF-MIR, 199 MIR-GENE, 93 MIR-TF hypomethylated regulatory pairs, and 195 TF–GENE, 67 TF-MIR, 108 MIR-GENE, 76 MIR-TF hypermethylated regulatory pairs (Table 1). The epigenetic network and the correlation among the genes, TFs, and miRNAs, are represented in Figure 4.

**Figure 5:**
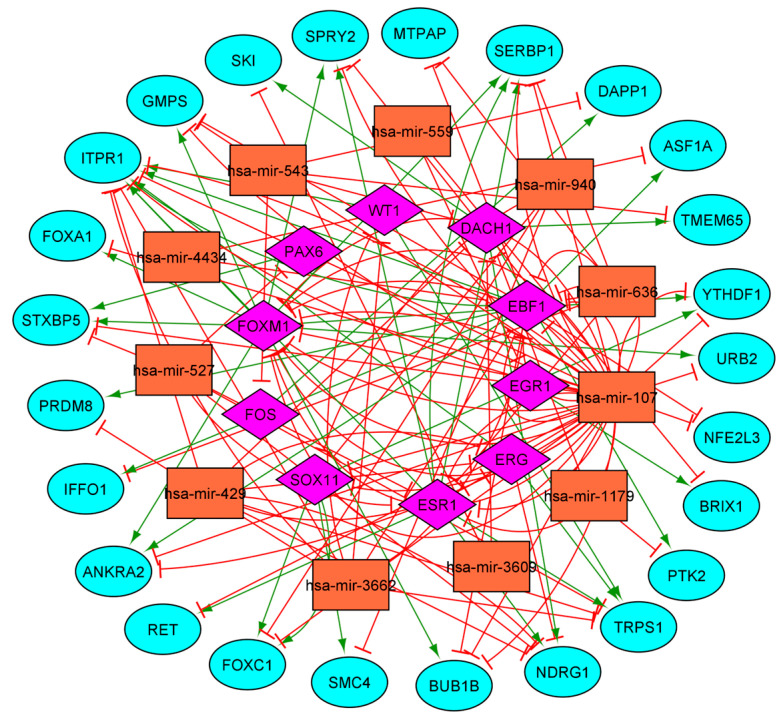
Epigenetic network of the 11 miRNA-mediated FFLs identified. Blue circles = genes; orange boxes = miRNAs; pink diamonds = TFs; green arrows = gene expression activation; red T shape arrows = gene expression inhibition.

Based on regulations of TFs and miRNAs to genes, the three types of three-node FFLs were identified: 110 TF-mediated FFL, 43 miRNA-mediated FFL, and five composite FFL (Table S4A–C), involving 18 hypermethylated and 32 hypomethylated genes, eight up-regulated and nine down-regulated TFs, and 21 up-regulated and seven down-regulated miRNAs. The TF-mediated FFLs comprised *AR*, *CBX2*, *E2F1*, *EBF1*, *EGR1*, *ERG*, *ESR1*, *FOS*, *FOXM1*, *MYBL2*, *PPARG*, *TAL1*, and *TEAD4* as the drivers, and the miR-107, miR-1179, miR-3609, miR-3662, miR-429, miR-4434, miR-527, miR-543, miRf-559, miR-636, and miR-940, were responsible for driving the miRNA-mediated FFLs (Figure 5). Among the regulatory interactions involved in the identified FFL types, 17 had experimental support based on validated miRNA–target interactions retrieved from the multiMiR/R (v.1.16.0) package, which integrates data from multiple curated databases.

**Figure 6:**
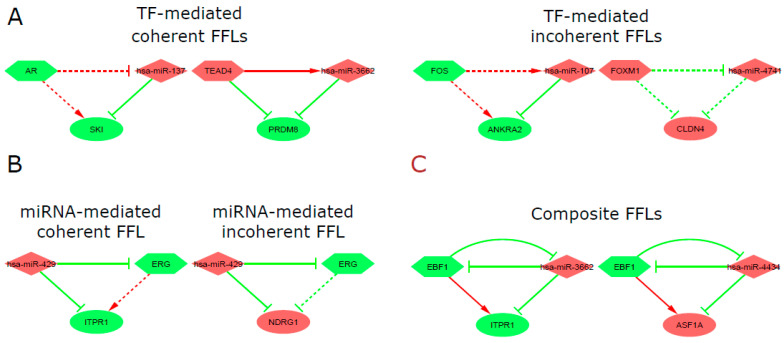
Representation of Feed-Forward Loops. (**A**) TF-mediated coherent and TF-mediated incoherent FFLs, (**B**) miRNA-mediated coherent and incoherent FFLs, and (**C**) composite FFLs. Nodes: diamonds = miRNAs; hexagons = TFs; ovals = genes; arrows = activation; T-shaped arrows = repression.

The types (TF, miRNA mediated coherent and incoherent, and composite FFLs) of three-node FFLs involving selected major regulators mentioned, such as the TFs, *AR*, *EBF1*, *FOS, ERG*, *FOXM1*, and *TEAD4*, and the miR-107, miR-137, miR-3662, miR-429, and miR-4434, are presented in Figure 6.

### 3.3. Identified Signaling Pathways and Gene Ontology Terms of the Hypo and Hypermethylated Probe–Gene Pairs, TFs, and miRNAs

**Figure 7:**
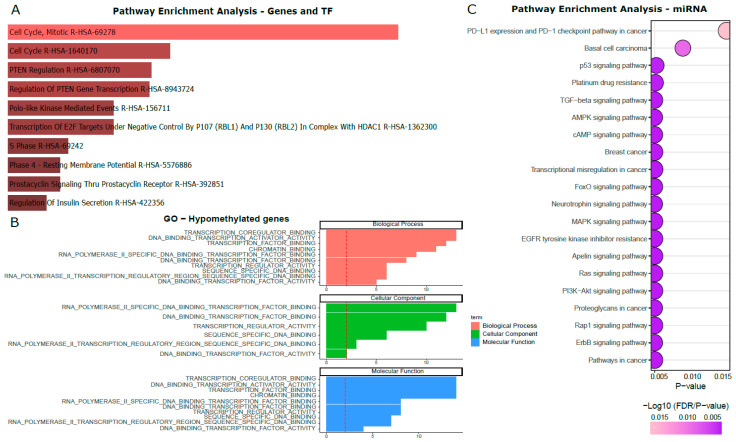
Pathway enrichment analysis and gene ontology (GO) analysis of hypomethylated genes upregulated miRNAs and TFs. (**A**) Top ten pathways (**B**) GO terms, and (**C**) top 20 pathways related to upregulated miRNAs.

**Figure 8:**
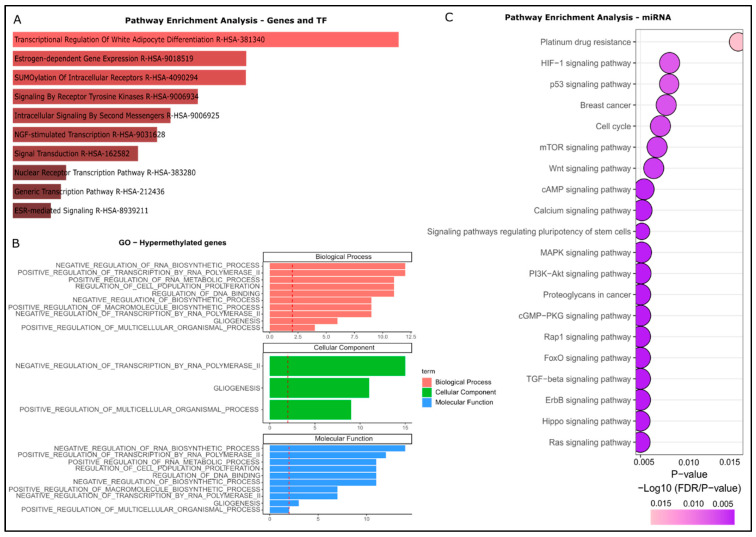
Pathway enrichment analysis and gene ontology (GO) analysis of hypermethylated genes upregulated miRNAs and TFs. (**A**) Top ten pathways (**B**) GO terms, and (**C**) top 20 pathways related to upregulated miRNAs.

To determine the biological pathways and processes influenced by the dysregulated methylation/expression of genes and TFs, GO and REACTOME pathway analyses were performed. In the group of the hypomethylated/upregulated genes and TFs, 84 significant REACTOME pathways (*p* < 0.05), and 96 GO terms (*p* < 0.05) were identified. Of the 96 GO terms, 50 were involved in the Biological Process, six in the Cellular Component, and 31 in Molecular Function. In the group of the hypermethylated/downregulated genes and TF, we identified 74 significant REACTOME pathways, and 27 GO terms, of which 11 were associated with Biological Process, three with Cellular Component, and 13 with Molecular Function (Table S5(A1–A3,B1–B3)). The GO terms were shown to be distinct according to the methylation gene status: in the hypomethylated set of genes, these terms were mostly related to binding activities and signaling pathways, such as cell cycle activities and PTEN regulation (Figure 7A,B). On the other hand, in the hypermethylated group, the terms identified were mostly related to the regulation of processes that include RNA and TF and signaling transcription pathways (Figure 8A,B).

Similarly, the pathway analysis of the miRNAs was performed using microT-CDS, TarBase, and TargetScan databases. The intersection of the three databases (*p* < 0.05) is presented in Figure 7C and Figure 8C for the group of the hypo/upregulation and hyper/downregulation genes, respectively. The pathways that were enriched in both groups were: breast cancer; platinum drug resistance; ErbB signaling pathway; Rap1 signaling pathway; FoxO signaling pathway; TGF-beta signaling pathway; proteoglycans in cancer; cAMP signaling pathway; p53 signaling pathway; and Ras signaling pathway. All these 20 pathways are associated with cancer, and involved in the process of drug resistance, cell proliferation, migration, differentiation, motility, apoptosis, epithelial–mesenchymal transition (EMT), angiogenesis, and metastasis.

### 3.4. Identified Hallmark Gene Set Process Categories of the Hypo and Hypermethylated Probe–Gene Pairs, TFs, and miRNAs

**Figure 9:**
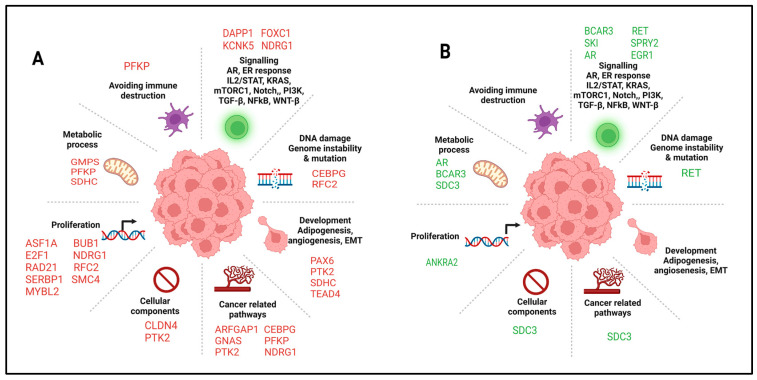
Process categories of the genes and TFs based on the Hallmark gene set collection of the MsigDB database analysis. (**A**) Hypomethylated genes and upregulated TFs (in red) (**B**) Hypermethylated genes and downregulated TFs (in green).

Pathway enrichment analysis was also performed using the Hallmark gene set collection through the MsigDB (v.2023.1) database. In the group of the hypomethylated/upregulated genes and TFs, the eight defined process categories were observed: cellular component, development, DNA damage, avoiding immune destruction, metabolic, cancer-related pathways, proliferation, and signaling (Figure 9A).

In the group of the hypermethylated/downregulated genes and TFs only six of the eight defined categories were observed (Figure 9B) (Table S6A,B). The same analysis using the miRNAs showed different categories according to the miRNA expression: six categories (development, DNA damage, metabolic, pathway, proliferation, and signaling) for the upregulated miRNAs and five (development, DNA damage, pathway, proliferation, and signaling) for the downregulated miRNAs.

## 4. Discussion

The dysregulation of the epigenetic machinery, at the distal cis-regulatory regions, has been shown to contribute to breast cancer development and progression [53]. However, there is limited information on how they regulate specific breast cancer subtypes, such as the BLBC.

The analysis of the distal methylated probes in samples of BLBC and non-tumor breast cancer retrieved from the TCGA database revealed 152 differentially methylated genes. Among these, 99 exhibited hypomethylation and 53 hypermethylation patterns in BLBC. A negative correlation was observed between the methylation status of these genes and their corresponding expression levels. The search for the enriched motifs belonging to the methylated probe–gene pairs identified revealed the involvement of several TFs in the formation of FFLs. Among these, TFs, *AR*, *EBF1*, *ERG*, *FOS*, *FOXM1*, and *TEAD4* were identified as major regulators of the FFLs.

In BLBC, the presence of the androgen receptor (AR) is a clinically significant factor, associated with an increase in disease-free survival. Conversely, the lack of AR expression increases the risk of recurrence, and metastasis [54,55,56]. In fact, about 57% to 80% of triple-negative breast cancer (TNBC) lack *AR* expression (known as QNBC) [57] and were shown to present more aggressive biological and clinical behavior [58,59]. However, *AR* is expressed in a subset of BLBC cases (~20–40%), defining the so-called “Luminal *AR* (LAR)” subtype of TNBC [60,61]. In this context, activation of the *AR*–miR-137–*SKI* FFL may drive tumor aggressiveness and proliferation, by different mechanisms, including resistance to TGF-β–mediated growth inhibition [62,63]. Within this observed FFL, *AR* presents dual roles, directly upregulating *SKI* while simultaneously downregulating miR-137, a well-characterized tumor-suppressor miRNA known to inhibit multiple oncogenes, including *SKI*, and to regulate key cancer-related processes such as epithelial–mesenchymal transition (EMT), stemness, and therapy resistance in breast cancer [64,65,66]. Together, these interactions can lead to a sustained elevation of *SKI* expression levels, reinforcing this oncogenic signaling in BLBC. Therapeutically, restoring miR-137 expression or inhibiting *AR* and *SKI* activity could disrupt this FFL and suppress tumor progression.

*EBF1* (Early B-cell Factor 1) is a transcription factor that was shown to contribute to cancer progression by negatively regulating the p53 signaling pathway or modulating telomerase reverse transcriptase expression [67,68]. In triple-negative breast cancer, it was observed to be highly expressed, in association with tumorigenicity and progression, acting on the autophagy and metabolism process [69]. MiR-3662 has also been implicated in triple-negative breast cancer, by targeting *HBP1*, a tumor suppressor that inhibits the Wnt/β-catenin signaling pathway. By suppressing *HBP1*, miR-3662 activates Wnt/β-catenin signaling, promoting tumor progression and metastasis [70]. The *ITPR1* (Inositol 1,4,5-trisphosphate receptor type 1) gene is a receptor that mediates calcium release from the endoplasmic reticulum, playing a role in cell proliferation and apoptosis. Lower expression of *ITPR1* has been associated with poor prognosis in breast cancer patients; it was related to the level of immune infiltration, particularly in patients with BLBC [71]. *EBF1* was also involved in an FFL with miR-4434 and *ASF1A*, a histone chaperone with a role in nucleosome assembly that impact DNA repair, replication, and transcription. In cancer cells, *ASF1A* inhibition has been shown to induce p53-dependent growth arrest and senescence of cancer cells [72]. While each of these FLL members play a role in cancer biology, a direct regulatory loop connecting all has not been documented. Our analysis suggests that *EBF1* may directly regulate the *ITPR1* and *ASF1A* genes, while also being involved in reciprocal negative regulation with the miRNAs, which in turn suppress the expression of these target genes.

The *ERG* transcription factor (*ETS*-related gene) is implicated in various cancers, where it plays a key role in maintaining endothelial homeostasis and promoting tumor angiogenesis [73]. MiR-429 is a member of the miR-200 family (miR-141, miR-200a, miR-200b, and miR-200c) and was reported to target the *ERG* gene in prostate cancer and promote angiogenesis in hepatocellular carcinoma [74]. This miRNA family is known to suppress proliferation, invasion, and epithelial–mesenchymal transition (EMT) in cancer cells [75]. In breast cancer, including the triple-negative subtype, miR-429, has been shown to regulate critical signaling pathways associated with cell progression [76,77,78]. The *ITPR1*, also observed in the above FFL, to the best of our knowledge, has no interaction with miR-429 or *ERG*. In contrast, members of the FFL formed by *ERG*-miR-429-*NDRG1* (*N-MYC* downstream regulated gene 1) have been reported to interact. A gene fusion between *NDRG1* and *ERG* has been identified in prostate cancer, resulting in a chimeric protein in ERG overexpressed prostate cancer cells [79]. The *NDRG1* functions as a suppressor of metastasis and its expression has been associated with tumor progression and prognosis in various cancers, including breast cancer [80]. However, similar to *ITPR1*, no direct association has been described between *NDRG1* and miR-429. Further studies are needed to determine the mechanisms and the biological relevance of the *ERG*-miR-429-*ITPR1* and *ERG*-miR-429-*NDRG1* regulatory FFLs.

*FOS*, a proto-oncogene responsible for encoding a nuclear DNA binding protein, plays a pivotal role in several types of cancer directly impacting signal transduction, cell differentiation, and proliferation [81]. In breast cancer, overexpression of *FOS* has been shown to suppress malignant phenotypes of breast cancer cells, suggesting its potential role as a tumor suppressor [82]. Studies demonstrated that *ANKRA2* is a direct transcriptional target of the tumor suppressor protein p53 indicating its role in p53 pathways [83]. Additionally, miR-107 is implicated in various cellular processes, including cell cycle and apoptosis [84,85]. The interplay between these molecules suggests that *FOS*-mir-107-*ANKRA2* may contribute to the suppression of tumor growth and progression through p53.

Overexpression of the *FOXM1* gene has been identified in several types of cancer, including breast cancer, in association with advanced tumor stage, high proliferation, tumor aneuploidy, poor survival, shorter relapse free-survival, and chemoresistance [86,87,88]. Claudin-4 (*CLDN4*) has been shown to be overexpressed in breast cancer and is associated with increased tumor aggressiveness and poor prognosis, particularly in TNBC [89,90,91]. Its overexpression enhances cell proliferation and invasion, partly by regulating signaling pathways such as *PAK4* and modulating the EMT. MiR-4741 has been pointed out as a potential biomarker related to chemoresistance [92]. In this context, the regulatory loop between *FOXM1*-miR-4741-*CLDN4* overexpression may cooperatively promote breast cancer aggressiveness and chemoresistance.

Finally, the *TEAD4* gene has also been found upregulated in various types of tumor tissues, where it has been shown to promote tumor growth and is associated with poor prognosis [93,94,95]. In the context of breast cancer, *TEAD4* functions as a key effector of the Hippo signaling pathway, a critical regulator of organ size, cell proliferation, and apoptosis. Additionally, the dysregulation of this pathway has been shown to support cancer stem cell maintenance, metastasis, and resistance to chemotherapy [96]. *PRDM5*, a known tumor suppressor and epigenetic regulator, is often silenced in breast cancer. It has been implicated in the repression of oncogenic pathways, including Wnt signaling, and its reduced expression is linked to enhanced stemness, EMT, and therapy resistance [97]. The direct interactions between *TEAD4*, *PRDM5*, and miR-3662 have not been previously studied, although their individual contributions to TNBC progression suggest potential crosstalk. Within this potential FFL, *TEAD4* may drive the expression of miR-3662, which in turn suppresses *PRDM5*, tipping the balance toward tumor-promoting gene expression. This triad could sustain a self-reinforcing loop of transcriptional activation and epigenetic deregulation in TNBC. Suggesting that the existence of a *TEAD4-*mir-3662-*PRDM5* FFL may promote aggressive behavior in TNBC.

Similarly to the regulatory influence exerted by TFs, DNA methylation plays an important role in the regulation of miRNAs contributing to cancer development [12,13,14,15,16,17,18]. In this study, we observed the alteration of expression levels of 500 miRNAs, including miR-107, miR-1179, miR-3609, miR-3662, miR-429, miR-4434, miR-527, miR-543, miR-559, miR-636, and miR-940, resultant of the distinctive patterns of the hypomethylated and hypermethylated distal probes. Specifically, the overexpression of miR-3662 has been shown to present antileukemic effects; in lung cancer, it is associated with advanced stages, positioning it as a potential biomarker of disease progression [98,99]. Conversely, miR-429, which was observed downregulated in BLBC, is involved in several types of cancer playing a role in modulating key biological processes such as epithelial–mesenchymal transition (EMT), cell invasion, metastasis, apoptosis, and drug resistance, revealing its impact on tumorigenesis [100].

Studies investigating the interaction of these transcription mechanisms, particularly the ones involved in FFLs, have provided valuable insights into the complex regulatory interactions between TFs and miRNAs [30,31,32]. In breast cancer, few studies, however, have constructed networks based on the interaction of TFs and ncRNAs [28,29,30,33]. In fact, in most of these studies, the constructed breast cancer networks were based on miRNAs and long non-coding RNAs (lncRNAs) [28,29,30,33].

LncRNAs are ncRNA with >200 nucleotides, that as miRNAs, can also act as oncogenes or tumor suppressors in cancer [101]. Beyond their regulatory impact at the transcriptional level, lncRNAs actively participate in epigenetic modifications, influencing the modification of histones and DNA methylation patterns [102,103]. This multifaceted involvement can impact gene expression by controlling the accessibility of genes (and miRNAs) to the transcriptional machinery. In this context, few lncRNAs have been found to act as competing endogenous RNAs (ceRNAs) to communicate with other RNA transcripts by competing miRNAs [104]. The study of Jiang et al. [33] investigated the regulations of TFs, miRNAs, and lncRNAs, identifying many FFLs for each breast cancer subtype. Each of these sub-networks was identified to target critical cancer pathways, including the ones involving cell cycle, and pathways in cancer. In the BLBC subtype, specifically, the authors highlighted the adhered junction pathway in which breast epithelial cells lose their adherent junction, a characteristic of the basal-like subtype.

In the enrichment analysis conducted in this study, the TFs and miRNAs identified as major regulators in the FFLs were shown to be involved in critical cancer-associated signaling pathways, such as the, ER, ERBB, FOXO, HIPPO, P53, PTEN RAP1, and TGF-beta pathways. Rather than focusing on the individual targets of these pathways, we emphasize their broader biological context, which are closely linked to hallmark features of BLBC, including drug resistance, epithelial–mesenchymal transition (EMT), metastatic potential. Importantly, these pathways align with the aggressive nature, poor clinical outcomes and limited therapeutic options associated with BLBC, underscoring their relevance as both mechanistic insights and potential avenues for therapeutic intervention. Interestingly, the involvement of these pathways, along with the GO terms identified, varied according to the methylation status of the distal probes. The additional enrichment analysis based on the hallmark gene set collection analysis underscored distinct biological process categories influenced by the genes’ methylation status. This comprehensive approach showed the interplay between epigenetic modifications and the intricate regulation of biological processes in the context of cancer development, particularly in BLBC. However, it is important to acknowledge the limitations of the study, including that it was based on computational predictions derived from publicly available datasets, particularly from TCGA, which may not fully represent the biological and clinical heterogeneity of BLBC, especially in underrepresented populations. While these computational predictions generate valuable hypotheses, experimental validation, through functional assays in well established in vitro and in vivo models, as well as studies incorporating longitudinal clinical data, is essential to confirm the biological relevance and potential clinical utility of the identified epigenetic interactions. Future research in these directions will be critical to assess the prognostic and therapeutic significance of these regulatory circuits in BLBC.

## 5. Conclusions

The results of this study highlight the non-random nature of the connection between alterations in DNA methylation and the dysregulation of the transcription cell machinery, including the regulatory action of genes, TFs, and miRNAs. These connections significantly affected key signaling pathways and biological processes associated with BLBC development and progression. This comprehensive regulatory network of FFLs not only reveals the intricate epigenetic machinery governing BLBC but also the promising opportunities for targeted therapeutic interventions and refined prognostic stratification strategies. However, further research is required to fully understand these mechanisms and indicate whether the identified epigenetic interactions can be of prognostic and/or therapeutic value for this breast cancer subtype.

## Figures and Tables

**Table 1:** Summary of the four types of regulatory relationships between genes, TFs, and miRNAs observed for the hypomethylated and hypermethylated probe–gene pairs.

	ENCODE/CHIP-X		multiMir	
	TF–GENE	TF-MIR	MIR-GENE	MIR-TF
Hypomethylated				
#Interaction	637	127	199	93
#gene	82	-	54	-
#miRNA	-	35	50	30
#TF	26	13	-	22
Hypermethylated				
#Interaction	195	67	108	76
#gene	40	-	35	-
#miRNA	-	23	37	24
#TF	21	12	-	20

## Data Availability

The datasets analyzed during the current study are available in The Cancer Genome Atlas—GDC Data Portal repository (https://portal.gdc.cancer.gov/projects/TCGA-BRCA (accessed on 10 April 2022)).

## Supplementary Materials

The following supporting information can be downloaded at: https://www.mdpi.com/article/10.3390/cells14161235/s1, Table S1A—Hypomethylated probe–gene pairs with the *p*-value (Pe), standard normal distribution *p*-value (raw.*p*), and location of the probe–gene pairs (sides right or left and distance) from the correlation between tumor samples and non-tumor samples; Table S1B—Hypermethylated probe–gene pairs with the *p*-value (Pe), standard normal distribution *p*-value (raw.*p*), and location of this probe–gene pair (sides Right or Left and distance) from the correlation between tumor samples and non-tumor samples; Table S2. Upregulated and downregulated miRNAs according to differential expression analysis from the correlation between tumor and non-tumor samples; Table S3A. Motifs and transcription factors lists associated with DNA hypomethylation; Table S3B. Motifs and transcription factors lists associated with DNA hypermethylation; Table S4A. FFLs mediated by the TFs identified, and the corresponding expression (red color, upregulated; green color, downregulated) and correlation (activation, repression) of the TFs, miRNAs (MIR) and genes; Table S4B. FFLs mediated by the miRNAs (MIR) identified, and the corresponding expression (red color, upregulated; green color, downregulated) and correlation (activation, repression) of the TFs, miRNAs and genes; Table S4C. Composite FFLs identified and the corresponding expression (red color, upregulated; green color, downregulated) and correlation (activation, repression) of the TF, miRNAs (MIR), and genes; Table S5A1. Hypomethylated genes and transcription factors MSigDB gene ontology biological process analysis; Table S5A2. Hypomethylated genes and transcription factors MSigDB gene ontology cellular component analysis; Table S5A3. Hypomethylated genes and transcription factors MSigDB gene ontology molecular function analysis; Table S5B1. Hypermethylated genes and transcription factors MSigDB gene ontology biological process analysis; Table S5B2. Hypermethylated genes and transcription factors MSigDB gene ontology cellular component analysis; Table S5B3. Hypermethylated genes and transcription factor MSigDB gene ontology molecular function analysis; Table S6A. Hallmarks of the gene set collection, their corresponding description and category of the differentially expressed genes composing the FFLs; Table S6B. Hallmarks of the gene set collection, their corresponding description and category of the differentially expressed transcription factors (TF) composing the FFLs.

## Abbreviations

The following abbreviations are used in this manuscript:

AR	Androgen Receptor
BLBC	Basal Like Breast Cancer
BP	Biological Process
BRCA	Breast Cancer
CC	Cellular Component
ceRNA	Competing endogenous RNAs
CpG	Cytosine–guanine dinucleotides
DE	Differentially expressed
ELMER	Enhancer Linking by Methylation/Expression Relationships
EMT	Epithelial–Mesenchymal Transition
FDA	Food and Drug Administration
FFL	Feed-Forward Loop
GO	Gene Ontology
KEGG	Kyoto Encyclopedia for Genes and Genomes
lncRNAs	Long non-coding RNAs
LogFC	Log2FoldChange
MF	Molecular Function
MiRNA	MicroRNA
MRTF	Master Regulator Transcription Factor
MSigDB	Molecular Signatures Database
PEA	Pathway Enrichment Analysis
SERM	Selective Estrogen Receptor Modulator
TCGA	The Cancer Genome Atlas
TF	Transcription Factor
TNBC	Triple-Negative Breast Cancer
