# Supplementary material for: Identification of Epigenetic Regulatory Networks of Gene Methylation–miRNA–Transcription Factor Feed-Forward Loops in Basal-like Breast Cancer
Source: Cells. 2025 Aug 10;14(16):1235. doi: 10.3390/cells14161235 (PMC12384104; doi:10.3390/cells14161235)
Supplement: Supplementary file 1 [file cells-14-01235-s001.zip › cells-3739841-supplementary.pdf]

Table S1A – Hypomethylated probe-gene pairs with the p-value (Pe), standard normal distribution p-value (raw.p), and location of the probe-gene pairs (sides right or left and distance) from the correlation between tumor samples and non-tumor samples.

Probe	GeneID	Symbol	Distance	Sides	Raw.p	Pe
cg04962865	ENSG00000205002	AARD	890465	R7	7,93E-04	1,66E+12
cg00144425	ENSG00000133627	ACTR3B	0	L1	1,42E-10	3,31E+10
cg12304937	ENSG00000105963	ADAP1	257522	R5	3,43E+07	3,65E+12
cg09966455	ENSG00000101199	ARFGAP1	-9122	L2	1,18E+01	8,29E+11
cg12098441	ENSG00000111875	ASF1A	0	L1	3,95E+06	6,96E+11
cg26655004	ENSG00000186009	ATP4B	789	L1	4,81E+07	3,31E+10
cg26230851	ENSG00000158850	B4GALT3	-83514	L10	4,42E-10	9,94E+10
cg05046589	ENSG00000095739	BAMBI	0	L1	5,35E+02	1,99E+12
cg16232234	ENSG00000113460	BRIX1	-321780	L5	3,26E+00	9,61E+11
cg27155939	ENSG00000156970	BUB1B	106059	R6	2,89E-11	1,33E+12
cg06579154	ENSG00000180066	C10orf91	-489270	L6	6,23E+00	5,97E+11
cg24699005	ENSG00000228300	C19orf24	83185	R8	9,94E+02	5,30E+11
cg04059696	ENSG00000173557	C2orf70	-409552	L10	5,33E+00	4,64E+11
cg26427498	ENSG00000253276	CCDC71L	307546	R4	5,44E+08	1,33E+12
cg09958065	ENSG00000151465	CDC123	-355937	L7	1,71E-08	7,29E+11
cg08365609	ENSG00000153879	CEBPG	137934	R9	6,63E+01	2,32E+11
cg15380291	ENSG00000159261	CLDN14	162368	R8	3,43E-05	2,32E+11
cg05418508	ENSG00000253958	CLDN23	-336896	L9	1,51E+08	3,31E+10
cg12620035	ENSG00000189143	CLDN4	-589	L1	2,28E+01	2,32E+11
cg17898069	ENSG00000168275	COA6	63711	R5	6,91E-09	3,98E+12
cg02733604	ENSG00000073067	CYP2W1	453398	R10	6,83E+08	7,96E+11
cg24690709	ENSG00000070190	DAPP1	0	L1	6,54E-03	1,66E+12
cg04207218	ENSG00000184029	DSCR4	-385581	L10	7,96E+08	9,28E+11
cg20821442	ENSG00000185055	EFCAB10	-38559	L2	3,39E+05	3,65E+12
cg04962865	ENSG00000147677	EIF3H	594396	R2	9,06E+02	5,97E+11
cg02130329	ENSG00000104412	EMC2	1084917	R9	9,31E+06	8,95E+11
cg10846615	ENSG00000163064	EN1	556	L1	8,35E-12	9,94E+10
cg01471264	ENSG00000163064	EN1	2330	L1	1,89E-05	2,65E+12
cg02327563	ENSG00000163064	EN1	2159	L1	1,06E-03	5,30E+11
cg26474124	ENSG00000035141	FAM136A	154648	R6	6,09E-07	4,31E+11
cg02039404	ENSG00000035141	FAM136A	154321	R6	1,39E-05	7,29E+11
cg21010202	ENSG00000054598	FOXC1	-1710	L1	1,96E-14	3,31E+10
cg18346398	ENSG00000054598	FOXC1	-1859	L1	1,98E-11	3,31E+10
cg01642550	ENSG00000141012	GALNS	-174948	L9	9,28E+00	4,97E+11
cg01218150	ENSG00000165474	GJB2	-198893	L4	2,94E-07	6,63E+11
cg18346398	ENSG00000112699	GMDS	8047	R1	3,40E-08	9,94E+10

cg21010202	ENSG00000112699	GMDS	8196	R1	4,39E-08	9,94E+10
cg20498563	ENSG00000163655	GMPS	142291	R6	6,30E-03	9,94E+11
cg10797197	ENSG00000087460	GNAS	0	L1	1,67E+00	1,99E+12
cg22531801	ENSG00000168243	GNG4	0	L1	4,02E+02	3,31E+10
cg26655004	ENSG00000185974	GRK1	19204	R1	3,42E+06	3,31E+10
cg12633410	ENSG00000081985	IL12RB2	0	L1	2,52E-02	4,97E+11
cg17898069	ENSG00000135750	KCNK1	-637230	L10	2,96E-02	5,30E+11
cg18705155	ENSG00000164626	KCNK5	0	L1	3,33E-06	7,96E+11
cg07587250	ENSG00000134248	LAMTOR5	213565	R10	5,87E+04	6,30E+11
cg19984355	ENSG00000153395	LPCAT1	-270155	L10	6,89E-07	3,31E+10
cg26878190	ENSG00000153395	LPCAT1	-270343	L10	5,80E-05	1,33E+12
cg08161205	ENSG00000171806	METTL18	-96700	L4	1,84E+04	4,31E+11
cg07626366	ENSG00000168303	MPLKIP	-764817	L5	8,45E-02	3,31E+10
cg26878190	ENSG00000171421	MRPL36	4077	L1	5,84E-06	3,31E+10
cg19984355	ENSG00000171421	MRPL36	4265	L1	2,06E-04	6,63E+10
cg00572560	ENSG00000107951	MTPAP	505211	R6	6,81E-02	6,63E+10
cg17667625	ENSG00000104419	NDRG1	24701	R1	1,29E+06	3,65E+12
cg24691330	ENSG00000147684	NDUFB9	400888	R14	1,82E-06	6,63E+10
cg26230851	ENSG00000158864	NDUFS2	-47087	L7	1,02E-05	6,63E+10
cg26878190	ENSG00000145494	NDUFS6	7092	R1	1,35E+00	9,94E+10
cg19984355	ENSG00000145494	NDUFS6	7280	R1	6,69E-04	2,98E+11
cg10797197	ENSG00000101158	NELFCD	112261	R5	6,31E+00	4,31E+11
cg07945582	ENSG00000050344	NFE2L3	0	L1	7,05E-13	3,31E+10
cg09958065	ENSG00000165609	NUDT5	-410382	L9	6,06E-09	3,65E+12
cg18659248	ENSG00000067057	PFKP	-499151	L9	5,80E-06	4,97E+11
cg23247845	ENSG00000067057	PFKP	-499174	L9	2,94E-05	8,62E+10
cg06271630	ENSG00000107959	PITRM1	-114731	L4	9,88E+03	9,94E+10
cg18813601	ENSG00000107959	PITRM1	-115537	L4	6,44E+05	2,98E+11
cg14534279	ENSG00000107959	PITRM1	-114932	L4	6,79E+05	3,31E+11
cg18659248	ENSG00000107959	PITRM1	-464028	L6	1,01E+07	3,31E+11
cg01277983	ENSG00000100344	PNPLA3	484056	R8	4,45E+09	8,95E+11
cg01676795	ENSG00000127948	POR	0	L1	2,65E-01	1,66E+12
cg15873474	ENSG00000101182	PSMA7	265789	R7	8,01E-08	6,96E+11
cg11546385	ENSG00000185627	PSMD13	-16390	L2	2,47E+02	6,63E+10
cg02563407	ENSG00000185627	PSMD13	-16483	L2	1,21E+03	6,63E+10
cg13185005	ENSG00000185627	PSMD13	-15965	L2	2,12E+04	4,31E+11
cg27109043	ENSG00000169398	PTK2	-235548	L6	8,71E+04	9,94E+10
cg05673731	ENSG00000060656	PTPRU	0	L1	4,49E+02	3,98E+12
cg20821442	ENSG00000091127	PUS7	-117163	L7	3,95E-03	3,98E+12
cg17588094	ENSG00000249859	PVT1	51507	R1	7,26E+04	6,96E+11

cg01393234	ENSG00000143811	PYCR2	22496	R2	1,31E+04	9,94E+10
cg04962865	ENSG00000164754	RAD21	798201	R4	3,02E-08	3,31E+10
cg12620035	ENSG00000049541	RFC2	398214	R10	2,95E-09	3,98E+12
cg08930413	ENSG00000106615	RHEB	-330830	L3	2,54E-07	2,32E+11
cg26230851	ENSG00000143252	SDHC	52772	R2	1,95E+03	4,64E+11
cg09958065	ENSG00000065665	SEC61A2	-436565	L10	3,51E+02	7,96E+11
cg10832107	ENSG00000142864	SERBP1	-28793	L1	4,16E+03	7,62E+11
cg12633410	ENSG00000142864	SERBP1	93359	R1	8,26E+02	8,62E+10
cg27109043	ENSG00000022567	SLC45A4	0	L1	4,60E+05	8,95E+11
cg04855961	ENSG00000113810	SMC4	397210	R7	6,45E-02	8,29E+11
cg26474124	ENSG00000143977	SNRPG	140035	R5	8,40E-09	7,96E+11
cg02039404	ENSG00000143977	SNRPG	139708	R5	1,07E-07	8,62E+10
cg19517912	ENSG00000142168	SOD1	157455	R4	6,61E+05	3,31E+10
cg12620035	ENSG00000106089	STX1A	-113610	L6	1,72E-04	9,94E+10
cg17898069	ENSG00000059588	TARBP1	81568	R6	1,83E+02	2,32E+11
cg24691330	ENSG00000147687	TATDN1	350272	R11	5,69E+00	5,97E+11
cg24691330	ENSG00000164983	TMEM65	167976	R7	1,13E+01	8,62E+10
cg26230851	ENSG00000158882	TOMM40L	-30736	L3	4,89E-01	3,98E+12
cg24691330	ENSG00000183665	TRMT12	312607	R8	1,40E+03	8,95E+11
cg04962865	ENSG00000104447	TRPS1	-238058	L3	4,61E+05	6,30E+11
cg21538013	ENSG00000135763	URB2	-56070	L3	4,17E+00	4,31E+11
cg04962865	ENSG00000147679	UTP23	718771	R3	4,55E+06	1,66E+12
cg16353508	ENSG00000149658	YTHDF1	111039	R7	4,17E+01	2,32E+11

Table S1B – Hypermethylated probe-gene pairs with the p-value (Pe), standard normal distribution p-value (raw.p), and location of this probe-gene pair (sides Right or Left and distance) from the correlation between tumor samples and non-tumor samples.

**Probe**	**GeneID**	**Symbol**	**Distance**	**Sides**	**Raw.p**	**Pe**
cg25136495	ENSG00000010295	IFFO1	-64468	L7	7,59E+00	9,59E+11
cg16215422	ENSG00000144891	AGTR1	1273905	R14	4,27E-12	6,94E+11
cg23147574	ENSG00000144891	AGTR1	1273467	R14	7,62E-12	8,27E+11
cg11795854	ENSG00000164331	ANKRA2	249050	R10	5,33E-04	5,95E+11
cg02495823	ENSG00000164331	ANKRA2	248872	R10	1,48E-04	8,27E+11

cg14780466	ENSG00000084674	APOB	330375	R5	2,31E-06	2,65E+11
cg02922879	ENSG00000137936	BCAR3	0	L1	2,78E+05	4,63E+12
cg18316974	ENSG00000189195	BTBD8	-296752	L9	1,49E+01	6,61E+11
cg07025650	ENSG00000177994	C2orf73	-99633	L2	9,64E-08	2,98E+11
cg21865150	ENSG00000136436	CALCOCO2	104109	R4	9,16E-10	4,30E+12
cg16557178	ENSG00000136436	CALCOCO2	104039	R4	9,99E-09	4,63E+12
cg23905164	ENSG00000136436	CALCOCO2	80835	R2	5,32E-08	8,93E+11
cg04573550	ENSG00000176435	CLEC14A	42993	R1	6,11E-11	7,60E+11
cg07152216	ENSG00000153002	CPB1	1367668	R15	6,53E-05	6,61E+11
cg08529882	ENSG00000121898	CPXM2	563265	R9	1,41E+02	8,60E+11
cg19029904	ENSG00000136160	EDNRB	-611954	L9	2,28E-11	1,98E+11
cg04932551	ENSG00000129514	FOXA1	0	L1	8,84E-01	2,98E+11
cg03736795	ENSG00000237125	HAND2-AS1	4112	R1	1,63E-02	4,96E+10
cg26311734	ENSG00000163106	HPGDS	464164	R4	1,39E-04	6,94E+11
cg11687330	ENSG00000080854	IGSF9B	825218	R7	3,44E-03	6,61E+11
cg07351192	ENSG00000150995	ITPR1	-248247	L8	7,37E-10	2,31E+12
cg21831931	ENSG00000150995	ITPR1	-248427	L8	1,47E-07	6,61E+11
cg09881545	ENSG00000150995	ITPR1	-248104	L8	3,47E-07	8,93E+11
cg07260592	ENSG00000213071	LPAL2	-158820	L3	9,75E-01	4,30E+12
cg06424065	ENSG00000013288	MAN2B2	329260	R4	1,32E+00	2,31E+12

cg06470822	ENSG00000189134	NKAPL	51813	R3	2,71E-14	4,30E+12
cg16733705	ENSG00000133636	NTS	600455	R5	1,90E-02	5,62E+11
cg09403666	ENSG00000223928	NUP35P1	985269	R8	1,27E+07	5,29E+10
cg15904283	ENSG00000165312	OTUD1	243518	R5	6,16E+01	9,92E+09
cg12337525	ENSG00000152784	PRDM8	-59744	L2	2,12E-08	6,61E+11
cg09645574	ENSG00000112619	PRPH2	-189046	L7	1,66E-02	9,92E+09
cg03171770	ENSG00000165731	RET	178787	R3	5,73E+09	6,61E+11
cg14836522	ENSG00000162512	SDC3	0	L1	4,31E+07	1,32E+12
cg12483545	ENSG00000157933	SKI	0	L1	5,94E+05	7,60E+11
cg03851159	ENSG00000165646	SLC18A2	109492	R6	1,67E+01	5,95E+11
cg27466845	ENSG00000168065	SLC22A11	-57386	L3	2,87E-01	2,65E+11
cg07260592	ENSG00000146477	SLC22A3	-226512	L4	1,52E-11	3,31E+11
cg22369786	ENSG00000136158	SPRY2	0	L1	1,27E-11	3,31E+11
cg04573550	ENSG00000139874	SSTR1	0	L1	2,35E-11	3,31E+11
cg03354508	ENSG00000164506	STXBP5	-57885	L3	3,14E+08	7,60E+11
cg12317414	ENSG00000166317	SYNPO2L	0	L1	6,75E-02	1,65E+12
cg17723653	ENSG00000166317	SYNPO2L	0	L1	3,93E+00	7,60E+11
cg06204711	ENSG00000092607	TBX15	0	L1	9,60E-08	9,26E+11
cg08445802	ENSG00000112837	TBX18	-8295	L1	1,07E+03	1,98E+11
cg26469608	ENSG00000089225	TBX5	878039	R10	4,35E+00	4,63E+12

cg09887059	ENSG0000018681 5	TPCN1	-180273	L7	3,15E-09	1,98E+11
cg09338032	ENSG0000018681 5	TPCN1	-180218	L7	5,81E-09	2,98E+11
cg05114739	ENSG0000023360 8	TWIST2	-406674	L6	9,07E-05	5,29E+10
cg03758150	ENSG0000019859 7	ZNF536	0	L1	4,80E+03	1,98E+11
cg23331421	ENSG0000019859 7	ZNF536	0	L1	5,62E+03	1,98E+11
cg23642130	ENSG0000019859 7	ZNF536	0	L1	5,67E+05	5,95E+11
cg05924485	ENSG0000021397 3	ZNF99	-23424	L1	7,48E-03	3,31E+11
cg10721834	ENSG0000021397 3	ZNF99	-23112	L1	1,11E+01	1,65E+12

Table S2. Upregulated and downregulated miRNAs according to differential expression analysis from the correlation between tumor and non-tumor samples.

miRNA	down/up-regulated
hsa-let-7a-1	downregulated
hsa-let-7a-2	downregulated
hsa-let-7a-3	downregulated
hsa-let-7b	downregulated
hsa-let-7c	downregulated
hsa-mir-100	downregulated
hsa-mir-101-1	downregulated
hsa-mir-101-2	downregulated
hsa-mir-105-1	upregulated
hsa-mir-105-2	upregulated
hsa-mir-106a	upregulated
hsa-mir-106b	upregulated
hsa-mir-107	upregulated
hsa-mir-10a	downregulated
hsa-mir-10b	downregulated
hsa-mir-1-1	downregulated
hsa-mir-1179	downregulated
hsa-mir-1180	upregulated
hsa-mir-1185-1	downregulated
hsa-mir-1-2	downregulated
hsa-mir-1224	upregulated

hsa-mir-1226	upregulated
hsa-mir-1228	downregulated
hsa-mir-1229	upregulated
hsa-mir-1247	downregulated
hsa-mir-1248	upregulated
hsa-mir-1249	downregulated
hsa-mir-1254-1	upregulated
hsa-mir-1254-2	upregulated
hsa-mir-1258	downregulated
hsa-mir-125a	downregulated
hsa-mir-125b-1	downregulated
hsa-mir-125b-2	downregulated
hsa-mir-126	downregulated
hsa-mir-1262	downregulated
hsa-mir-1269a	upregulated
hsa-mir-1269b	upregulated
hsa-mir-127	downregulated
hsa-mir-1270	upregulated
hsa-mir-1276	upregulated
hsa-mir-1277	upregulated
hsa-mir-128-1	upregulated
hsa-mir-128-2	upregulated
hsa-mir-1283-1	upregulated
hsa-mir-1283-2	upregulated
hsa-mir-1284	upregulated
hsa-mir-1288	upregulated
hsa-mir-1292	upregulated
hsa-mir-1293	upregulated
hsa-mir-1294	downregulated
hsa-mir-1295a	downregulated
hsa-mir-1295b	upregulated
hsa-mir-1301	upregulated
hsa-mir-1304	upregulated
hsa-mir-1307	upregulated
hsa-mir-130b	upregulated
hsa-mir-133a-1	downregulated
hsa-mir-133a-2	downregulated
hsa-mir-133b	downregulated
hsa-mir-134	downregulated
hsa-mir-135a-1	downregulated
hsa-mir-135a-2	downregulated
hsa-mir-135b	upregulated
hsa-mir-137	upregulated
hsa-mir-138-1	upregulated
hsa-mir-138-2	upregulated
hsa-mir-139	downregulated
hsa-mir-141	upregulated
hsa-mir-142	upregulated

hsa-mir-143	downregulated
hsa-mir-144	downregulated
hsa-mir-145	downregulated
hsa-mir-146a	upregulated
hsa-mir-146b	upregulated
hsa-mir-147b	upregulated
hsa-mir-148a	upregulated
hsa-mir-148b	upregulated
hsa-mir-149	downregulated
hsa-mir-150	upregulated
hsa-mir-151b	downregulated
hsa-mir-153-1	downregulated
hsa-mir-153-2	downregulated
hsa-mir-1537	upregulated
hsa-mir-1538	upregulated
hsa-mir-154	downregulated
hsa-mir-155	upregulated
hsa-mir-15a	upregulated
hsa-mir-15b	upregulated
hsa-mir-17	upregulated
hsa-mir-181a-1	upregulated
hsa-mir-181b-1	upregulated
hsa-mir-181b-2	upregulated
hsa-mir-181c	upregulated
hsa-mir-181d	upregulated
hsa-mir-182	upregulated
hsa-mir-183	upregulated
hsa-mir-187	upregulated
hsa-mir-188	upregulated
hsa-mir-18a	upregulated
hsa-mir-18b	upregulated
hsa-mir-190a	downregulated
hsa-mir-190b	downregulated
hsa-mir-1910	upregulated
hsa-mir-1914	upregulated
hsa-mir-1915	upregulated
hsa-mir-192	upregulated
hsa-mir-193a	downregulated
hsa-mir-193b	downregulated
hsa-mir-195	downregulated
hsa-mir-196a-1	upregulated
hsa-mir-196a-2	upregulated
hsa-mir-19a	upregulated
hsa-mir-19b-1	upregulated
hsa-mir-19b-2	upregulated
hsa-mir-200a	upregulated
hsa-mir-200b	upregulated
hsa-mir-200c	upregulated

hsa-mir-202	downregulated
hsa-mir-203a	upregulated
hsa-mir-203b	upregulated
hsa-mir-204	downregulated
hsa-mir-205	downregulated
hsa-mir-20a	upregulated
hsa-mir-20b	upregulated
hsa-mir-21	upregulated
hsa-mir-210	upregulated
hsa-mir-211	downregulated
hsa-mir-2110	downregulated
hsa-mir-2114	upregulated
hsa-mir-2115	downregulated
hsa-mir-215	downregulated
hsa-mir-218-1	downregulated
hsa-mir-218-2	downregulated
hsa-mir-219a-1	upregulated
hsa-mir-219b	upregulated
hsa-mir-22	downregulated
hsa-mir-2277	upregulated
hsa-mir-26a-1	downregulated
hsa-mir-26a-2	downregulated
hsa-mir-26b	downregulated
hsa-mir-27a	upregulated
hsa-mir-28	downregulated
hsa-mir-296	downregulated
hsa-mir-299	downregulated
hsa-mir-29a	downregulated
hsa-mir-29c	downregulated
hsa-mir-301a	upregulated
hsa-mir-301b	upregulated
hsa-mir-3065	upregulated
hsa-mir-3074	upregulated
hsa-mir-30a	downregulated
hsa-mir-31	upregulated
hsa-mir-3117	upregulated
hsa-mir-3127	upregulated
hsa-mir-3129	downregulated
hsa-mir-3136	upregulated
hsa-mir-3140	upregulated
hsa-mir-3145	upregulated
hsa-mir-3150b	upregulated
hsa-mir-3157	downregulated
hsa-mir-3161	upregulated
hsa-mir-3170	upregulated
hsa-mir-3174	upregulated
hsa-mir-3176	upregulated
hsa-mir-3187	upregulated

hsa-mir-3194	upregulated
hsa-mir-3199-1	downregulated
hsa-mir-3199-2	downregulated
hsa-mir-32	upregulated
hsa-mir-3200	upregulated
hsa-mir-320b-1	upregulated
hsa-mir-320b-2	upregulated
hsa-mir-320c-1	downregulated
hsa-mir-320d-1	upregulated
hsa-mir-323a	downregulated
hsa-mir-324	upregulated
hsa-mir-326	downregulated
hsa-mir-328	downregulated
hsa-mir-329-1	downregulated
hsa-mir-329-2	downregulated
hsa-mir-330	upregulated
hsa-mir-331	upregulated
hsa-mir-335	downregulated
hsa-mir-337	downregulated
hsa-mir-33a	upregulated
hsa-mir-33b	upregulated
hsa-mir-340	upregulated
hsa-mir-345	upregulated
hsa-mir-3609	upregulated
hsa-mir-3610	upregulated
hsa-mir-3613	upregulated
hsa-mir-3614	upregulated
hsa-mir-3615	upregulated
hsa-mir-3618	upregulated
hsa-mir-3619	upregulated
hsa-mir-362	upregulated
hsa-mir-3620	upregulated
hsa-mir-363	downregulated
hsa-mir-3651	upregulated
hsa-mir-3652	upregulated
hsa-mir-365a	downregulated
hsa-mir-365b	downregulated
hsa-mir-3662	upregulated
hsa-mir-3664	upregulated
hsa-mir-3677	upregulated
hsa-mir-3680-1	upregulated
hsa-mir-3682	upregulated
hsa-mir-369	downregulated
hsa-mir-3690-1	upregulated
hsa-mir-3691	upregulated
hsa-mir-370	downregulated
hsa-mir-374b	downregulated
hsa-mir-376a-1	downregulated

hsa-mir-376a-2	downregulated
hsa-mir-376b	downregulated
hsa-mir-376c	downregulated
hsa-mir-377	downregulated
hsa-mir-378a	downregulated
hsa-mir-378c	downregulated
hsa-mir-378d-1	downregulated
hsa-mir-378d-2	downregulated
hsa-mir-379	downregulated
hsa-mir-380	downregulated
hsa-mir-381	downregulated
hsa-mir-382	downregulated
hsa-mir-3911	upregulated
hsa-mir-3917	upregulated
hsa-mir-3922	upregulated
hsa-mir-3926-1	downregulated
hsa-mir-3926-2	downregulated
hsa-mir-3928	upregulated
hsa-mir-3934	upregulated
hsa-mir-3940	upregulated
hsa-mir-3942	upregulated
hsa-mir-3944	upregulated
hsa-mir-409	downregulated
hsa-mir-410	downregulated
hsa-mir-411	downregulated
hsa-mir-412	downregulated
hsa-mir-421	upregulated
hsa-mir-429	upregulated
hsa-mir-431	downregulated
hsa-mir-432	downregulated
hsa-mir-4326	upregulated
hsa-mir-433	downregulated
hsa-mir-4431	upregulated
hsa-mir-4433b	downregulated
hsa-mir-4434	upregulated
hsa-mir-4442	upregulated
hsa-mir-4443	upregulated
hsa-mir-4444-1	upregulated
hsa-mir-4444-2	upregulated
hsa-mir-4449	upregulated
hsa-mir-4473	upregulated
hsa-mir-4482	upregulated
hsa-mir-4491	downregulated
hsa-mir-4501	upregulated
hsa-mir-4517	upregulated
hsa-mir-451a	downregulated
hsa-mir-452	downregulated
hsa-mir-4522	upregulated

hsa-mir-4524a	downregulated
hsa-mir-4529	downregulated
hsa-mir-454	upregulated
hsa-mir-455	upregulated
hsa-mir-4638	upregulated
hsa-mir-4640	upregulated
hsa-mir-4645	upregulated
hsa-mir-4652	upregulated
hsa-mir-4658	upregulated
hsa-mir-4661	upregulated
hsa-mir-4664	upregulated
hsa-mir-4665	upregulated
hsa-mir-4668	upregulated
hsa-mir-4674	upregulated
hsa-mir-4677	upregulated
hsa-mir-4678	downregulated
hsa-mir-4680	upregulated
hsa-mir-4687	upregulated
hsa-mir-4701	downregulated
hsa-mir-4709	downregulated
hsa-mir-4724	upregulated
hsa-mir-4726	upregulated
hsa-mir-4732	downregulated
hsa-mir-4741	upregulated
hsa-mir-4742	upregulated
hsa-mir-4745	upregulated
hsa-mir-4746	upregulated
hsa-mir-4758	upregulated
hsa-mir-4766	upregulated
hsa-mir-4772	upregulated
hsa-mir-4777	upregulated
hsa-mir-4784	upregulated
hsa-mir-4786	upregulated
hsa-mir-4787	upregulated
hsa-mir-4791	upregulated
hsa-mir-4797	upregulated
hsa-mir-483	downregulated
hsa-mir-485	downregulated
hsa-mir-486-1	downregulated
hsa-mir-486-2	downregulated
hsa-mir-487a	downregulated
hsa-mir-487b	downregulated
hsa-mir-488	downregulated
hsa-mir-491	downregulated
hsa-mir-494	downregulated
hsa-mir-495	downregulated
hsa-mir-496	downregulated
hsa-mir-497	downregulated

hsa-mir-4999	upregulated
hsa-mir-5001	upregulated
hsa-mir-5002	upregulated
hsa-mir-5003	upregulated
hsa-mir-500a	upregulated
hsa-mir-500b	upregulated
hsa-mir-501	upregulated
hsa-mir-5010	downregulated
hsa-mir-502	upregulated
hsa-mir-503	upregulated
hsa-mir-505	upregulated
hsa-mir-5090	upregulated
hsa-mir-5092	upregulated
hsa-mir-511	downregulated
hsa-mir-512-1	upregulated
hsa-mir-512-2	upregulated
hsa-mir-514a-1	upregulated
hsa-mir-514a-2	upregulated
hsa-mir-514a-3	upregulated
hsa-mir-516a-1	upregulated
hsa-mir-516a-2	upregulated
hsa-mir-518a-1	upregulated
hsa-mir-518a-2	upregulated
hsa-mir-518e	upregulated
hsa-mir-519a-1	upregulated
hsa-mir-519a-2	upregulated
hsa-mir-519c	upregulated
hsa-mir-520b	upregulated
hsa-mir-520e	upregulated
hsa-mir-520f	upregulated
hsa-mir-521-1	upregulated
hsa-mir-521-2	upregulated
hsa-mir-522	upregulated
hsa-mir-525	upregulated
hsa-mir-527	upregulated
hsa-mir-532	upregulated
hsa-mir-539	downregulated
hsa-mir-541	downregulated
hsa-mir-543	downregulated
hsa-mir-545	upregulated
hsa-mir-548au	upregulated
hsa-mir-548aw	downregulated
hsa-mir-548b	upregulated
hsa-mir-548d-1	upregulated
hsa-mir-548d-2	upregulated
hsa-mir-548e	upregulated
hsa-mir-548f-1	upregulated
hsa-mir-548k	upregulated

hsa-mir-548o-2	upregulated
hsa-mir-548q	downregulated
hsa-mir-548s	upregulated
hsa-mir-548v	upregulated
hsa-mir-549a	upregulated
hsa-mir-551a	upregulated
hsa-mir-551b	downregulated
hsa-mir-552	upregulated
hsa-mir-556	upregulated
hsa-mir-5571	upregulated
hsa-mir-5581	upregulated
hsa-mir-5582	upregulated
hsa-mir-5586	upregulated
hsa-mir-559	upregulated
hsa-mir-561	downregulated
hsa-mir-5683	downregulated
hsa-mir-5684	upregulated
hsa-mir-5687	downregulated
hsa-mir-5690	upregulated
hsa-mir-5694	downregulated
hsa-mir-5695	upregulated
hsa-mir-5699	upregulated
hsa-mir-570	upregulated
hsa-mir-573	upregulated
hsa-mir-574	downregulated
hsa-mir-576	upregulated
hsa-mir-577	upregulated
hsa-mir-579	upregulated
hsa-mir-580	upregulated
hsa-mir-582	upregulated
hsa-mir-585	downregulated
hsa-mir-588	upregulated
hsa-mir-590	upregulated
hsa-mir-592	upregulated
hsa-mir-597	upregulated
hsa-mir-605	downregulated
hsa-mir-607	downregulated
hsa-mir-6125	upregulated
hsa-mir-616	upregulated
hsa-mir-624	downregulated
hsa-mir-632	upregulated
hsa-mir-636	upregulated
hsa-mir-639	upregulated
hsa-mir-642a	downregulated
hsa-mir-643	upregulated
hsa-mir-6501	upregulated
hsa-mir-6503	downregulated
hsa-mir-6507	downregulated

hsa-mir-6509	upregulated
hsa-mir-651	upregulated
hsa-mir-6510	downregulated
hsa-mir-6511b-1	downregulated
hsa-mir-6511b-2	downregulated
hsa-mir-6513	downregulated
hsa-mir-6514	downregulated
hsa-mir-6516	upregulated
hsa-mir-653	downregulated
hsa-mir-654	downregulated
hsa-mir-655	downregulated
hsa-mir-656	downregulated
hsa-mir-658	upregulated
hsa-mir-659	upregulated
hsa-mir-660	upregulated
hsa-mir-664a	downregulated
hsa-mir-664b	downregulated
hsa-mir-665	downregulated
hsa-mir-671	upregulated
hsa-mir-6715a	downregulated
hsa-mir-6715b	downregulated
hsa-mir-6716	downregulated
hsa-mir-6720	upregulated
hsa-mir-6728	upregulated
hsa-mir-6730	downregulated
hsa-mir-6731	upregulated
hsa-mir-6737	upregulated
hsa-mir-6744	upregulated
hsa-mir-6746	downregulated
hsa-mir-675	downregulated
hsa-mir-676	downregulated
hsa-mir-6761	downregulated
hsa-mir-6766	downregulated
hsa-mir-6781	upregulated
hsa-mir-6783	upregulated
hsa-mir-6793	upregulated
hsa-mir-6797	upregulated
hsa-mir-6798	upregulated
hsa-mir-6802	downregulated
hsa-mir-6803	downregulated
hsa-mir-6807	upregulated
hsa-mir-6809	downregulated
hsa-mir-6814	upregulated
hsa-mir-6815	upregulated
hsa-mir-6827	downregulated
hsa-mir-6832	upregulated
hsa-mir-6833	downregulated
hsa-mir-6834	upregulated

hsa-mir-6837	downregulated
hsa-mir-6842	upregulated
hsa-mir-6844	upregulated
hsa-mir-6850	upregulated
hsa-mir-6852	upregulated
hsa-mir-6854	upregulated
hsa-mir-6875	upregulated
hsa-mir-6883	downregulated
hsa-mir-6891	upregulated
hsa-mir-6892	downregulated
hsa-mir-7-1	upregulated
hsa-mir-7112	upregulated
hsa-mir-7156	upregulated
hsa-mir-7-2	upregulated
hsa-mir-7-3	upregulated
hsa-mir-758	downregulated
hsa-mir-760	upregulated
hsa-mir-765	upregulated
hsa-mir-766	upregulated
hsa-mir-767	upregulated
hsa-mir-769	upregulated
hsa-mir-7705	upregulated
hsa-mir-7706	upregulated
hsa-mir-7974	upregulated
hsa-mir-7976	downregulated
hsa-mir-8072	upregulated
hsa-mir-873	upregulated
hsa-mir-874	downregulated
hsa-mir-877	upregulated
hsa-mir-885	upregulated
hsa-mir-889	downregulated
hsa-mir-891a	downregulated
hsa-mir-9-1	upregulated
hsa-mir-9-2	upregulated
hsa-mir-92a-1	upregulated
hsa-mir-92b	upregulated
hsa-mir-93	upregulated
hsa-mir-9-3	upregulated
hsa-mir-934	upregulated
hsa-mir-935	downregulated
hsa-mir-937	upregulated
hsa-mir-939	upregulated
hsa-mir-940	upregulated
hsa-mir-942	upregulated
hsa-mir-96	upregulated
hsa-mir-98	upregulated
hsa-mir-99a	downregulated

Table S3A. Motifs and transcription factors lists associated with DNA hypomethylation.

https://docs.google.com/spreadsheets/d/1ZpACmyO_54jseUdjQ9A8t92qzDcOOXmdc-xgOXz540E/edit?usp=drive_link

Table S3B. Motifs and transcription factors lists associated with DNA hypermethylation.

https://docs.google.com/spreadsheets/d/1IRKqpibLyTABr97RBUZoFkuRbBzui7BzVjyt12aAeDU/edit?usp=drive_link

Table S4A. FFLs mediated by the TFs identified, and the corresponding expression (red color, upregulated; green color, downregulated) and correlation (activation, repression) of the TFs, miRNAs (MIR) and genes.

TF-mediated FFLs					
**TF**	**MIR**	**GENE**	**TF-MIR**	**TF-GENE**	**MIR-GENE**
AR	hsa-mir-137	KCNK1	repression	activation	repression
AR	hsa-mir-137	SKI	repression	activation	repression
AR	hsa-mir-137	SMC4	repression	activation	repression
CBX2	hsa-mir-429	ITPR1	activation	activation	repression
E2F1	hsa-mir-4741	CLDN4	repression	activation	repression
E2F1	hsa-mir-4784	IGSF9B	activation	activation	repression
E2F1	hsa-mir-636	IFFO1	repression	activation	repression
E2F1	hsa-mir-636	MTPAP	repression	activation	repression
E2F1	hsa-mir-636	SERBP1	repression	activation	repression
E2F1	hsa-mir-639	GNAS	activation	activation	repression
E2F1	hsa-mir-760	FAM136A	activation	activation	repression
EBF1	hsa-mir-3652	BAMBI	repression	activation	repression
EBF1	hsa-mir-3652	MAN2B2	repression	activation	repression
EBF1	hsa-mir-3652	RFC2	repression	repression	repression
EBF1	hsa-mir-3662	ITPR1	repression	activation	repression
EBF1	hsa-mir-3662	PRDM8	repression	activation	repression
EBF1	hsa-mir-429	ITPR1	repression	activation	repression
EBF1	hsa-mir-429	RAD21	repression	activation	repression
EBF1	hsa-mir-4434	ASF1A	repression	activation	repression
EBF1	hsa-mir-4784	IGSF9B	repression	activation	repression
EBF1	hsa-mir-636	IFFO1	repression	activation	repression
EBF1	hsa-mir-636	MTPAP	repression	repression	repression
EBF1	hsa-mir-760	FAM136A	repression	repression	repression
EBF1	hsa-mir-765	ACTR3B	repression	repression	repression
EGR1	hsa-mir-2110	CLDN4	activation	repression	repression
EGR1	hsa-mir-3176	PFKP	activation	activation	repression
EGR1	hsa-mir-3652	BAMBI	activation	repression	repression
EGR1	hsa-mir-3652	MAN2B2	activation	activation	repression
EGR1	hsa-mir-3652	RFC2	activation	activation	repression

EGR1	hsa-mir-429	ITPR1	activation	activation	repression
EGR1	hsa-mir-429	NDRG1	activation	repression	repression
EGR1	hsa-mir-429	RAD21	activation	activation	repression
EGR1	hsa-mir-429	TBX5	activation	activation	repression
EGR1	hsa-mir-4434	ASF1A	repression	activation	repression
EGR1	hsa-mir-4741	CLDN4	activation	repression	repression
EGR1	hsa-mir-4784	IGSF9B	activation	activation	repression
EGR1	hsa-mir-636	IFFO1	repression	activation	repression
EGR1	hsa-mir-636	SERBP1	repression	activation	repression
EGR1	hsa-mir-639	GNAS	activation	repression	repression
EGR1	hsa-mir-760	FAM136A	activation	activation	repression
ERG	hsa-mir-548q	TRPS1	activation	activation	repression
ESR1	hsa-mir-190b	SDC3	repression	activation	repression
FOS	hsa-mir-107	ANKRA2	activation	activation	repression
FOS	hsa-mir-107	BUB1B	activation	activation	repression
FOS	hsa-mir-107	FOXA1	activation	activation	repression
FOS	hsa-mir-107	FOXC1	activation	repression	repression
FOS	hsa-mir-107	ITPR1	activation	activation	repression
FOS	hsa-mir-107	NFE2L3	activation	repression	repression
FOS	hsa-mir-1179	PTK2	activation	repression	repression
FOS	hsa-mir-1276	CEBPG	activation	repression	repression
FOS	hsa-mir-190b	RHEB	repression	repression	repression
FOS	hsa-mir-3609	EIF3H	activation	repression	repression
FOS	hsa-mir-3609	FOXC1	activation	repression	repression
FOS	hsa-mir-3652	BAMBI	repression	repression	repression
FOS	hsa-mir-3652	RFC2	repression	repression	repression
FOS	hsa-mir-421	FOXC1	activation	repression	repression
FOS	hsa-mir-421	OTUD1	activation	activation	repression
FOS	hsa-mir-4434	ASF1A	repression	repression	repression
FOS	hsa-mir-4443	FOXC1	repression	repression	repression
FOS	hsa-mir-4784	IGSF9B	activation	activation	repression
FOS	hsa-mir-496	ANKRA2	repression	activation	repression
FOS	hsa-mir-496	BCAR3	repression	activation	repression
FOS	hsa-mir-496	FOXA1	repression	activation	repression
FOS	hsa-mir-496	GMDS	repression	repression	repression
FOS	hsa-mir-496	ITPR1	repression	activation	repression
FOS	hsa-mir-496	SDHC	repression	repression	repression
FOS	hsa-mir-636	IFFO1	repression	activation	repression
FOS	hsa-mir-760	FAM136A	activation	repression	repression
FOS	hsa-mir-935	IFFO1	repression	activation	repression
FOXM1	hsa-mir-3652	RFC2	repression	activation	repression
FOXM1	hsa-mir-3652	SYNPO2L	repression	activation	repression
FOXM1	hsa-mir-3662	ITPR1	repression	activation	repression
FOXM1	hsa-mir-3662	PRDM8	repression	activation	repression
FOXM1	hsa-mir-3662	SYNPO2L	repression	activation	repression
FOXM1	hsa-mir-3662	TPCN1	repression	activation	repression
FOXM1	hsa-mir-4434	ASF1A	repression	activation	repression
FOXM1	hsa-mir-4741	CLDN4	repression	repression	repression

FOXM1	hsa-mir-636	MTPAP	repression	activation	repression
FOXM1	hsa-mir-636	SERBP1	repression	activation	repression
FOXM1	hsa-mir-760	FAM136A	repression	activation	repression
MYBL2	hsa-mir-2110	CLDN4	repression	activation	repression
MYBL2	hsa-mir-3652	RFC2	repression	activation	repression
MYBL2	hsa-mir-4434	ASF1A	repression	activation	repression
MYBL2	hsa-mir-4741	CLDN4	repression	activation	repression
MYBL2	hsa-mir-4784	CYP2W1	activation	activation	repression
MYBL2	hsa-mir-636	SERBP1	repression	activation	repression
MYBL2	hsa-mir-639	GNAS	repression	activation	repression
MYBL2	hsa-mir-760	FAM136A	repression	activation	repression
PPARG	hsa-mir-760	FAM136A	activation	activation	repression
TAL1	hsa-mir-1248	NUDT5	activation	activation	repression
TAL1	hsa-mir-3662	ITPR1	repression	activation	repression
TAL1	hsa-mir-3662	TBX18	repression	activation	repression
TAL1	hsa-mir-3662	TPCN1	repression	activation	repression
TAL1	hsa-mir-4434	ASF1A	activation	activation	repression
TAL1	hsa-mir-636	IFFO1	repression	activation	repression
TAL1	hsa-mir-636	MTPAP	repression	activation	repression
TAL1	hsa-mir-636	SERBP1	repression	activation	repression
TAL1	hsa-mir-639	GNAS	activation	activation	repression
TAL1	hsa-mir-760	FAM136A	repression	activation	repression
TEAD4	hsa-mir-1248	NUDT5	repression	activation	repression
TEAD4	hsa-mir-137	KCNK1	repression	activation	repression
TEAD4	hsa-mir-2110	CLDN4	repression	activation	repression
TEAD4	hsa-mir-3176	PFKP	repression	activation	repression
TEAD4	hsa-mir-3652	BAMBI	repression	activation	repression
TEAD4	hsa-mir-3662	PRDM8	activation	repression	repression
TEAD4	hsa-mir-4434	ASF1A	activation	activation	repression
TEAD4	hsa-mir-4741	CLDN4	repression	activation	repression
TEAD4	hsa-mir-4784	IGSF9B	activation	activation	repression
TEAD4	hsa-mir-636	IFFO1	repression	repression	repression
TEAD4	hsa-mir-760	FAM136A	activation	activation	repression

Table S4B. FFLs mediated by the miRNAs (MIR) identified, and the corresponding expression (red color, upregulated; green color, downregulated) and correlation (activation, repression) of the TFs, miRNAs and genes.

MIR-mediated FFL					
MIR	TF	GENE	MIR-TF	MIR-GENE	TF-GENE
hsa-mir-107	EBF1	ANKRA2	repression	repression	activation
hsa-mir-107	EBF1	BUB1B	repression	repression	repression
hsa-mir-107	EBF1	GMPS	repression	repression	repression
hsa-mir-107	EBF1	ITPR1	repression	repression	activation
hsa-mir-107	EBF1	NFE2L3	repression	repression	repression

hsa-mir-107	ESR1	ITPR1	repression	repression	activation
hsa-mir-107	ESR1	RET	repression	repression	activation
hsa-mir-107	ESR1	SERBP1	repression	repression	activation
hsa-mir-107	ESR1	SPRY2	repression	repression	activation
hsa-mir-107	FOXM1	ANKRA2	repression	repression	activation
hsa-mir-107	FOXM1	BUB1B	repression	repression	activation
hsa-mir-107	FOXM1	FOXA1	repression	repression	activation
hsa-mir-107	FOXM1	GMPS	repression	repression	activation
hsa-mir-107	FOXM1	ITPR1	repression	repression	activation
hsa-mir-107	FOXM1	SERBP1	repression	repression	activation
hsa-mir-107	FOXM1	SPRY2	repression	repression	activation
hsa-mir-107	FOXM1	STXBP5	repression	repression	activation
hsa-mir-107	FOXM1	URB2	repression	repression	activation
hsa-mir-107	FOXM1	YTHDF1	repression	repression	activation
hsa-mir-107	SOX11	FOXC1	repression	repression	activation
hsa-mir-107	SOX11	YTHDF1	repression	repression	activation
hsa-mir-1179	DACH1	PTK2	repression	repression	activation
hsa-mir-3609	DACH1	SKI	repression	repression	activation
hsa-mir-3662	EBF1	ITPR1	repression	repression	activation
hsa-mir-3662	EBF1	PRDM8	repression	repression	activation
hsa-mir-3662	WT1	ITPR1	repression	repression	activation
hsa-mir-3662	WT1	TRPS1	repression	repression	activation
hsa-mir-429	DACH1	NDRG1	repression	repression	activation
hsa-mir-429	ERG	ITPR1	repression	repression	activation
hsa-mir-429	ERG	NDRG1	repression	repression	repression
hsa-mir-429	ERG	TRPS1	repression	repression	activation
hsa-mir-4434	EBF1	ASF1A	repression	repression	activation
hsa-mir-527	ESR1	NDRG1	repression	repression	activation
hsa-mir-527	ESR1	TRPS1	repression	repression	activation
hsa-mir-527	PAX6	STXBP5	repression	repression	activation
hsa-mir-543	DACH1	TMEM65	repression	repression	activation
hsa-mir-543	FOS	DAPP1	repression	repression	activation
hsa-mir-559	EGR1	BRIX1	repression	repression	activation
hsa-mir-636	EBF1	IFFO1	repression	repression	activation
hsa-mir-636	EBF1	MTPAP	repression	repression	repression
hsa-mir-636	ESR1	SERBP1	repression	repression	activation
hsa-mir-940	SOX11	FOXC1	repression	repression	activation
hsa-mir-940	SOX11	SMC4	repression	repression	activation

Table S4C. Composite FFLs identified and the corresponding expression (red color, upregulated; green color, downregulated) and correlation (activation, repression) of the TF, miRNAs (MIR), and genes.

Composite FFL						
**MIR**	**TF**	**GENE**	**MIR-TF**	**MIR-GENE**	**TF-MIR**	**TF-GENE**
hsa-mir-3662	EBF1	PRDM8	repression	repression	repression	activation
hsa-mir-3662	EBF1	ITPR1	repression	repression	repression	activation

hsa-mir-4434	EBF1	ASF1A	repression	repression	repression	activation
hsa-mir-636	EBF1	IFFO1	repression	repression	repression	activation
hsa-mir-636	EBF1	MTPAP	repression	repression	repression	repression

Table S5A1. Hypomethylated genes and transcription factors MSigDB gene ontology biological process analysis.

https://docs.google.com/spreadsheets/d/119lhzsdnpFxPPNAUJ7Io8AlWWZjWlA_V/edit?usp=sharing&ouid=105448875743515672438&rtpof=true&sd=true

Table S5A2. Hypomethylated genes and transcription factors MSigDB gene ontology cellular component analysis.

https://docs.google.com/spreadsheets/d/12ETElwflhz8JO35Kle81UAo0RFT7bQ0A/edit?usp=drive_link&ouid=105448875743515672438&rtpof=true&sd=true

Table S5A3. Hypomethylated genes and transcription factors MSigDB gene ontology molecular function analysis.

https://docs.google.com/spreadsheets/d/17TQJ7a2QutBJKaqNa85rHbqUas9TQ-ma/edit?usp=drive_link&ouid=105448875743515672438&rtpof=true&sd=true

Table S5B1. Hypermethylated genes and transcription factors MSigDB gene ontology biological process analysis.

https://docs.google.com/spreadsheets/d/1odGSalCHBbFtUI8au-vAWDQhjgcz3UEo/edit?usp=drive_link&ouid=105448875743515672438&rtpof=true&sd=true

Table S5B2. Hypermethylated genes and transcription factors MSigDB gene ontology cellular component analysis.

https://docs.google.com/spreadsheets/d/1V1Vs7Sp5OffoWu0IKgd-9_8V08wLGWPG/edit?usp=drive_link&ouid=105448875743515672438&rtpof=true&sd=true

Table S5B3. Hypermethylated genes and transcription factor MSigDB gene ontology molecular function analysis.

https://docs.google.com/spreadsheets/d/1NltZzR_UNr5I_DsMWb2Bihoxj3fUrqLB/edit?usp=drive_link&ouid=105448875743515672438&rtpof=true&sd=true

Table S6A. Hallmarks of the gene set collection, their corresponding description and category of the differentially expressed genes composing the FFLs.

GENE	Hallmarks	Description	Category
Upregulated / hypomethylated genes			
ARFGAP1	HALLMARK_UNFOLDED_PROTEIN_RESPONSE	Genes up-regulated during unfolded protein response, a cellular stress response related to the endoplasmic reticulum.	pathway
ASF1A	HALLMARK_E2F_TARGETS	Genes encoding cell cycle related targets of E2F transcription factors.	proliferation
BUB1B	HALLMARK_E2F_TARGETS	Genes encoding cell cycle related targets of E2F transcription factors.	proliferation
CEBPG	HALLMARK_UNFOLDED_PROTEIN_RESPONSE	Genes up-regulated during unfolded protein response, a cellular stress response related to the endoplasmic reticulum.	pathway
	HALLMARK_UV_RESPONSE_UP	Genes up-regulated in response to ultraviolet (UV) radiation.	DNA damage
CLDN4	HALLMARK_APICAL_JUNCTION	Genes encoding components of apical junction complex.	cellular component
DAPP1	HALLMARK_MTORC1_SIGNALING	Genes up-regulated through activation of mTORC1 complex.	signaling
	HALLMARK_PI3K_AKT_MTOR_SIGNALING	Genes up-regulated by activation of the PI3K/AKT/mTOR pathway.	signaling
FOXC1	HALLMARK_ESTROGEN_RESPONSE_EARLY	Genes defining early response to estrogen.	signaling
	HALLMARK_ESTROGEN_RESPONSE_LATE	Genes defining late response to estrogen.	signaling
GMPS	HALLMARK_HEME_METABOLISM	Genes involved in metabolism of heme (a cofactor consisting of iron and porphyrin) and erythroblast differentiation.	metabolic
	HALLMARK_MTORC1_SIGNALING	Genes up-regulated through activation of mTORC1 complex.	signaling

GNAS	HALLMARK_PROTEIN_SECRETION	Genes involved in protein secretion pathway.	pathway
KCNK5	HALLMARK_ESTROGEN_RESPONSE_EARLY	Genes defining early response to estrogen.	signaling
	HALLMARK_ESTROGEN_RESPONSE_LATE	Genes defining late response to estrogen.	signaling
NDRG1	HALLMARK_ANDROGEN_RESPONSE	Genes defining response to androgens.	signaling
	HALLMARK_HYPOXIA	Genes up-regulated in response to low oxygen levels (hypoxia).	pathway
	HALLMARK_IL2_STAT5_SIGNALING	Genes up-regulated by STAT5 in response to IL2 stimulation.	signaling
	HALLMARK_P53_PATHWAY	Genes involved in p53 pathways and networks.	proliferation
PFKP	HALLMARK_GLYCOLYSIS	Genes encoding proteins involved in glycolysis and gluconeogenesis.	metabolic
	HALLMARK_HYPOXIA	Genes up-regulated in response to low oxygen levels (hypoxia).	pathway
	HALLMARK_INTERFERON_GAMMA_RESPONSE	Genes up-regulated in response to IFNG [GeneID=3458].	immune
	HALLMARK_REACTIVE_OXYGEN_SPECIES_PATHWAY	Genes up-regulated by reactive oxigen species (ROS).	pathway
PTK2	HALLMARK_ADIPOGENESIS	Genes up-regulated during adipocyte differentiation (adipogenesis).	development
	HALLMARK_APICAL_JUNCTION	Genes encoding components of apical junction complex.	cellular component
	HALLMARK_APOPTOSIS	Genes mediating programmed cell death (apoptosis) by activation of caspases.	pathway
RAD21	HALLMARK_E2F_TARGETS	Genes encoding cell cycle related targets of E2F transcription factors.	proliferation
	HALLMARK_G2M_CHECKPOINT	Genes involved in the G2/M checkpoint, as in progression through the cell division cycle.	proliferation
RFC2	HALLMARK_DNA_REPAIR	Genes involved in DNA repair.	DNA damage

	HALLMARK_E2F_TARGETS	Genes encoding cell cycle related targets of E2F transcription factors.	proliferation
SDHC	HALLMARK_ADIPOGENESIS	Genes up-regulated during adipocyte differentiation (adipogenesis).	development
	HALLMARK_FATTY_ACID_METABOLISM	Genes encoding proteins involved in metabolism of fatty acids.	metabolic
	HALLMARK_GLYCOLYSIS	Genes encoding proteins involved in glycolysis and gluconeogenesis.	metabolic
	HALLMARK_OXIDATIVE_PHOSPHORYLATION	Genes encoding proteins involved in oxidative phosphorylation.	metabolic
SERBP1	HALLMARK_MYC_TARGETS_V1	A subgroup of genes regulated by MYC - version 1 (v1).	proliferation
SMC4	HALLMARK_E2F_TARGETS	Genes encoding cell cycle related targets of E2F transcription factors.	proliferation
	HALLMARK_G2M_CHECKPOINT	Genes involved in the G2/M checkpoint, as in progression through the cell division cycle.	proliferation
	HALLMARK_MITOTIC_SPINDLE	Genes important for mitotic spindle assembly.	proliferation
SNRPG	HALLMARK_MYC_TARGETS_V1	A subgroup of genes regulated by MYC - version 1 (v1).	proliferation
Downregulated / hypermethylated genes			
ANKRA2	HALLMARK_P53_PATHWAY	Genes involved in p53 pathways and networks.	proliferation
BCAR3	HALLMARK_BILE_ACID_METABOLISM	Genes involve in metabolism of bile acids and salts.	metabolic
	HALLMARK_TGF_BETA_SIGNALING	Genes up-regulated in response to TGFB1 [GeneID=7040].	signaling
RET	HALLMARK_ESTROGEN_RESPONSE_EARLY	Genes defining early response to estrogen.	signaling
	HALLMARK_ESTROGEN_RESPONSE_LATE	Genes defining late response to estrogen.	signaling
	HALLMARK_UV_RESPONSE_UP	Genes up-regulated in response to ultraviolet (UV) radiation.	DNA damage

SDC3	HALLMARK_APICAL_JUNCTION	Genes encoding components of apical junction complex.	cellular component
	HALLMARK_GLYCOLYSIS	Genes encoding proteins involved in glycolysis and gluconeogenesis.	metabolic
	HALLMARK_HYPOXIA	Genes up-regulated in response to low oxygen levels (hypoxia).	pathway
SKI	HALLMARK_TGF_BETA_SIGNALING	Genes up-regulated in response to TGFB1 [GeneID=7040].	signaling
SPRY2	HALLMARK_KRAS_SIGNALING_UP	Genes up-regulated by KRAS activation.	signaling

Table S6B. Hallmarks of the gene set collection, their corresponding description and category of the differentially expressed transcription factors (TF) composing the FFLs.

**TF**	**Hallmarks**	**Description**	**Category**
Upregulated transcription fator			
E2F1	HALLMARK_G2M_CHECKPOINT	Genes involved in the G2/M checkpoint, as in progression through the cell division cycle.	proliferation
	HALLMARK_PI3K_AKT_MTOR_SIGNALING	Genes up-regulated by activation of the PI3K/AKT/mTOR pathway.	signaling
MYBL2	HALLMARK_E2F_TARGETS	Genes encoding cell cycle related targets of E2F transcription factors.	proliferation
	HALLMARK_G2M_CHECKPOINT	Genes involved in the G2/M checkpoint, as in progression through the cell division cycle.	proliferation
PAX6	HALLMARK_PANCREAS_BETA_CELLS	Genes specifically up-regulated in pancreatic beta cells.	development
TEAD4	HALLMARK_MYOGENESIS	Genes involved in development of skeletal muscle (myogenesis).	development
Downregulated transcription fator			
AR	HALLMARK_BILE_ACID_METABOLISM	Genes involve in metabolism of bile acids and salts.	metabolic

	HALLMARK_ESTROGEN_RESPONSE_EARLY	Genes defining early response to estrogen.	signaling
EGR1	HALLMARK_TNFA_SIGNALING_VIA_NFKB	Genes regulated by NF-kB in response to TNF [GeneID=7124].	signaling
ESR1	HALLMARK_XENOBIOTIC_METABOLISM	Genes encoding proteins involved in processing of drugs and other xenobiotics.	metabolic
FOS	HALLMARK_ESTROGEN_RESPONSE_EARLY	Genes defining early response to estrogen.	signaling
	HALLMARK_ESTROGEN_RESPONSE_LATE	Genes defining late response to estrogen.	signaling
	HALLMARK_HYPOXIA	Genes up-regulated in response to low oxygen levels (hypoxia).	pathway
	HALLMARK_P53_PATHWAY	Genes involved in p53 pathways and networks.	proliferation
	HALLMARK_TNFA_SIGNALING_VIA_NFKB	Genes regulated by NF-kB in response to TNF [GeneID=7124].	signaling
	HALLMARK_UV_RESPONSE_UP	Genes up-regulated in response to ultraviolet (UV) radiation.	DNA damage
	HALLMARK_P53_PATHWAY	Genes involved in p53 pathways and networks.	proliferation
	HALLMARK_TNFA_SIGNALING_VIA_NFKB	Genes regulated by NF-kB in response to TNF [GeneID=7124].	signaling
PPARG	HALLMARK_ADIPOGENESIS	Genes up-regulated during adipocyte differentiation (adipogenesis).	development
	HALLMARK_CHOLESTEROL_HOMEOSTASIS	Genes involved in cholesterol homeostasis.	metabolic
	HALLMARK_UV_RESPONSE_DN	Genes down-regulated in response to ultraviolet (UV) radiation.	DNA damage
TAL1	HALLMARK_HEME_METABOLISM	Genes involved in metabolism of heme (a cofactor consisting of iron and porphyrin) and erythroblast differentiation.	metabolic
